# Investigating the role of blood models in predicting rupture status of intracranial aneurysms

**DOI:** 10.1088/2057-1976/adcc34

**Published:** 2025-04-24

**Authors:** Zonghan Lyu, Mostafa Rezaeitaleshmahalleh, Nan Mu, Jingfeng Jiang

**Affiliations:** 1 Biomedical Engineering, Michigan Technological University, Houghton, MI, United States of America; 2 Joint Center for Biocomputing and Digital Health, Health Research Institute and Institute of Computing and Cybernetics, Michigan Technological University, Houghton, MI, United States of America

**Keywords:** hemodynamics, non-Newtonian, machine learning

## Abstract

**Purpose.:**

Selecting patients with high-risk intracranial aneurysms (IAs) is of clinical importance. Recent work in machine learning-based (ML) predictive modeling has demonstrated that lesion-specific hemodynamics within IAs can be combined with other information to provide critical insights for assessing rupture risk. However, how the adoption of blood rheology models (i.e., Newtonian and Non-Newtonian blood models) may influence ML-based predictive modeling of IA rupture risk has not been investigated.

**Methods and Materials.:**

In this study, we conducted transient CFD simulations using Newtonian and non-Newtonian rheology (Carreau-Yasuda [CY]) models on a large cohort of ‘patient-specific’ IA geometries (>100) under pulsatile flow conditions to investigate how each blood model may affect the characterization of the IAs’ rupture status. Key hemodynamic parameters were analyzed and compared, including wall shear stress (WSS) and vortex-based parameters. In addition, velocity-informatics features extracted from the flow velocity were utilized to train a support vector machine (SVM) model for rupture status prediction.

**Results.:**

Our findings demonstrate significant differences between the two models (i.e., Newtonian versus CY) regarding the WSS-related metrics. In contrast, the parameters derived from the flow vortices and velocity informatics agree. Similar to other studies, using a non-Newtonian CY model results in lower peak WSS and higher oscillatory shear index (OSI) values. Furthermore, integrating velocity informatics and machine learning achieved robust performance for both blood models (area under the curve [AUC] ^>^0.85).

**Conclusions.:**

Our preliminary study found that ML-based rupture status prediction derived from velocity informatics and geometrical parameters yielded comparable results despite differences observed in aneurysmal hemodynamics using two blood rheology models (i.e., Newtonian versus CY).

## 1. Introduction

Intracranial aneurysms (IAs) are pathological dilations of the cerebral arteries, afflicting an estimated 3%–5% of the global population (Vlak *et al* 2011). Although IAs are generally asymptomatic, aneurysm rupture leading to subarachnoid hemorrhage poses a severe health risk to patients. Since hemodynamics plays a vital role in the natural history of IAs (Sforza *et al* 2009, Sunderland *et al* 2022), considerable research efforts have been devoted to incorporating ‘patient-specific’ hemodynamics for more accurately assessing the risk of IA rupture. Recent advancements in computational fluid dynamics (CFD) have enabled ‘patient-specific’ modeling based on three-dimensional (3D) neurovascular imaging, allowing the characterization of *in vivo* intra-aneurysmal hemodynamic metrics (Steinman, 2002, Sunderland and Jiang, 2019, Chen *et al* 2020, Rezaeitaleshmahalleh *et al* 2023c), which cannot be achieved with conventional neurovascular imaging alone.

Predicting IAs’ rupture risk or status of IAs using machine learning (ML) algorithms has recently gained significant traction. Most relevant studies involving the computation of hemodynamics for IA rupture assessments have adopted a Newtonian fluid model (Xiang *et al* 2011, Detmer *et al* 2018b, Cho *et al* 2020, Tanioka *et al* 2020, Chen *et al* 2022, Mu *et al* 2023, Rezaeitaleshmahalleh *et al* 2023c). Notably, a small fraction of these studies used a non-Newtonian fluid model or did not specify an adopted flow model (Amigo *et al* 2021, Shi *et al* 2021).

In Newtonian models, blood flow is assumed to become smoother with increasing flow velocity and shear strain rate, resulting in constant viscosity. However, human blood is known to behave as a non-Newtonian fluid *in vivo* with shear-thinning properties (Nader *et al* 2019). Although the gross aneurysmal flow patterns remained comparable to those in non-Newtonian fluid models, when the Newtonian fluid model was used, previous studies have also demonstrated differences in the estimations of wall shear stress (WSS) and its derivatives (e.g., oscillatory shear index [OSI]) (Bernsdorf and Wang, 2009, Hippelheuser *et al* 2014, Oliveira *et al* 2021, Hosseini *et al* 2022, Yi *et al* 2022). Given the findings of these previous studies, the **primary question** of how the blood rheology model (i.e., Newtonian versus non-Newtonian) may influence the ML-based prediction of IAs’ rupture status or risk prediction has not received much attention. This unanswered question motivated the present study. Our **secondary objective** is to contribute to the growing need to understand the sensitivity of computed aneurysmal hemodynamics. Previous studies comparing Newtonian and non-Newtonian fluid models have been conducted using a small number of IAs (Bernsdorf and Wang, 2009, Hippelheuser *et al* 2014, Oliveira *et al* 2021, Hosseini *et al* 2022, Yi *et al* 2022). Thus, a systematic study involving a large number of IAs (>100), similar to ours, contributes to the archived literature. These computed hemodynamic markers are particularly important for characterizing the IA’s rupture status.

To this end, we aimed to investigate the differences in the CFD-calculated hemodynamics between Newtonian and non-Newtonian fluid models. In particular, we incorporated ML techniques to examine how hemodynamic features calculated using Newtonian and non-Newtonian (Carreau-Yasuda [CY]) fluid models may affect the evaluation of IA rupture status. Moreover, we employed velocity informatics (Rezaeitaleshmahalleh *et al* 2023b, 2023c, Rezaeitaleshmahalleh *et al* 2025), representing gross aneurysmal hemodynamics to quantify flow disturbances in addition to WSS and its derivatives. We also compared three non-Newtonian models (CY, Casson, and Herschel-Bulkley [HB]) in a subset of our data. Our study is the first to evaluate Newtonian and non-Newtonian blood rheology models using textural analysis of CFD-calculated flow data and to connect these findings with ML-based predictions of IAs’ rupture status.

## 2. Methods and materials

### 2.1. Dataset

The patient data for this study were obtained from three sources: the University of Michigan Medical Center (USA), Changhai Hospital in Shanghai (China), and the Aneurisk open-source repository (http://ecm2.mathcs.emory.edu/aneuriskweb/index). The inclusion criteria were as follows: (1) image quality sufficient for segmentation, morphological analysis, and CFD modeling; (2) IA sizes ranging from 4 to 25 mm with known rupture status; (3) absence of a nearby second IA; and (4) IA located in the anterior circulation. A total of 112 cerebral aneurysms (44 ruptured and 68 unruptured) were identified, all of which were saccular: 39 in the intracranial internal carotid artery (ICA), 52 in the middle cerebral artery (MCA), and 21 in the anterior cerebral artery (ACA). The rupture status of the IAs was obtained from the medical records.

**Figure 1.**
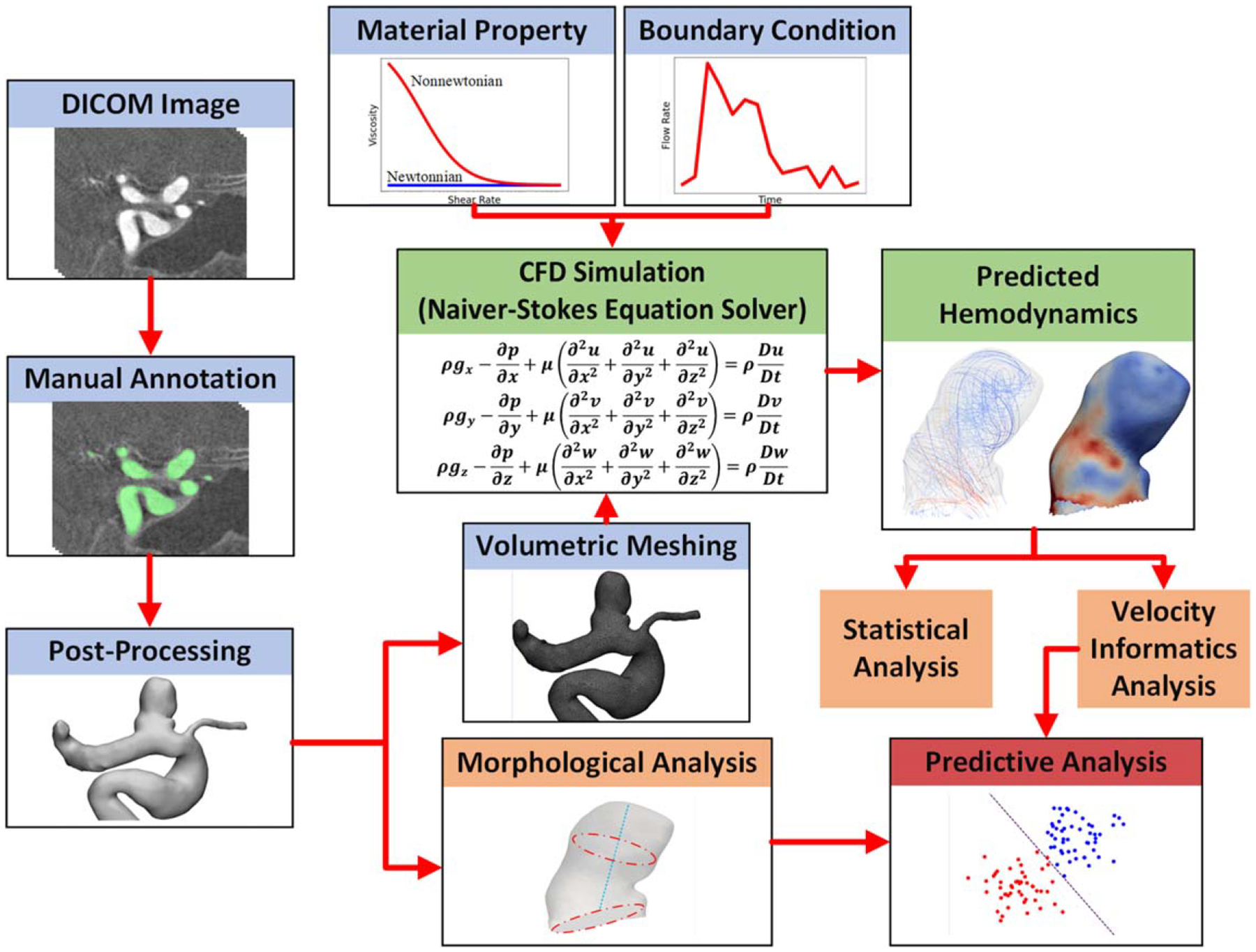
A flow chart showing the general workflow for acquiring hemodynamic parameters and data analytics

Figure 1 illustrates the overall workflow of this study, which comprises four major steps: (1) manual image annotation and volumetric meshing (shown in blue); (2) patient-specific CFD for hemodynamic parameters (shown in green); (3) morphological analysis and advanced statistical analysis (shown in orange); and (4) statistical and machine learning (ML)-based predictive analysis (shown in red).

### 2.2. Manual annotation and morphological analysis

The ‘patient-specific’ vessel structure reconstruction begins with manual annotation of the DICOM images. The image sets were initially imported into the Mimics Innovation Suite (v24.0, Materialise Inc., Leuven, Belgium) to create a mask representing the aneurysmal vasculature based on the pixel intensity differences induced by imaging contrast. Subsequently, the mask was converted into a stereolithography (STL) file and imported into 3-Matic software (Version 16.0, Materialise Inc., Leuven, Belgium) to address surface irregularities and achieve minimal smoothing while preserving the surface contour, meeting the fundamental requirements for CFD simulation. All vessel structures included the petrous-cervical region of the ICA as the inlet; thus, we could utilize a generalized blood flow waveform.

Subsequently, a morphological analysis was conducted on the isolated aneurysm structure. Key geometrical parameters, such as volume and height, as well as the Voronoi diagram characteristics (VDC), were calculated following the detailed procedures outlined in our previous publications (Sunderland *et al* 2021, Rezaeitaleshmahalleh *et al* 2023c), and Supplementary Materials (see section S2).

An open-source mesh generator, known as Tetgen, integrated into VMTK (Antiga *et al* 2008) was employed to generate computational meshes incorporating five boundary layers. Depending on the complexity and size of the vasculature, the resulting volumetric mesh consisted of approximately 1.5–3 million tetrahedral elements, with an average mesh size of $0.0022\;{mm}^{3}$.

### 2.3. Patient-specific CFD simulation

After generating the mesh, the commercial CFD simulation solver ANSYS FLUENT (v20.0, Ansys Inc., PA, USA) was employed to simulate blood flow by numerically solving the Navier–Stokes equations. Blood was assumed to be an incompressible, laminar, and isothermal fluid. For such a fluid, the continuity and Navier–Stokes equations are as follows:

(1) ∇⋅v=0

(2) $$\rho \left(v\cdot \nabla \right)v=-\nabla \;p+u\;\nabla^{2}\;v+\rho g$$

where v is the velocity, ρ is the fluid density, p is the pressure, g is the gravitational acceleration vector, and u is dynamic viscosity. The spatial discretization of pressure and momentum was accomplished using second-order and second-order upwind schemes, respectively, whereas temporal discretization utilized a second-order implicit scheme. The pressure-velocity coupling was resolved using the classic SIMPLE algorithm.

For fluid properties, the Newtonian model assumed blood to have a density of 1050 kg·m s^−1^ and a viscosity of 0.004 Pa·s. The Carreau-Yasuda (CY) model was applied in FLUENT for non-Newtonian behaviors, explicitly following the formulation derived by Bernabeu *et al* (Bernabeu *et al* 2013). This shear rate-dependent viscosity $\left(\eta (\dot{\gamma })\right)$ is calculated based on a strain-rate tensor multiplier (λ), power law index (n), and limiting zero and infinite shear rate viscosities ($\eta_{0}$ and $\eta_{\infty }$, respectively). The CY model can be expressed as follows:

(3) $$\eta (\dot{\gamma })=\eta_{\infty }+\left(\eta_{0}-\eta_{\infty }\right){\left[1+(\lambda \dot{\gamma }{)}^{a}\right]}^{(n-1)/a}$$

where $\eta_{0}=0.16Pa\cdot s,\;\eta_{\infty }=0.004Pa\cdot S,\;\lambda =8.2s,\;a=0.64$, and n=0.2128.

Because of the unavailability of *in vivo* patient-specific flow waveforms, an averaged pulsatile flow waveform, measured in healthy human subjects using magnetic resonance imaging (Gwilliam *et al* 2009), was used as the inlet boundary condition. Based on the average inlet diameter (0.0061m) of our dataset, the maximum Reynolds number was 830 at peak systole (9.05mL/s) under infinite shear rate viscosity (0.0035Pa⋅s). A zero-pressure boundary condition was applied to all the outlets. The CFD simulation was conducted with a time step size of 0.001 s. Four cardiac cycles over four seconds were simulated, and 20 constant-interval data points from the last cardiac cycle were saved and used for further data analysis. The convergence criterion was set to 10^−4^ for the three velocity components and continuity in the Fluent solver. We verified that these set convergence criteria were satisfied for all CFD simulations.

The above-mentioned CFD workflow has been verified with flow imaging in a pre-clinical canine aneurysm model, i.e., phase-contrast MRA (PC-MRA) (Jiang *et al* 2011a, Jain *et al* 2016) and ultrasound Doppler (Jiang *et al* 2011b).

### 2.4. Hemodynamic analysis and velocity informatics

In this study, all analyses were confined to isolated aneurysms, excluding the larger vasculature. Following the completion of the CFD simulation, wall shear stress (WSS) parameters were processed, including the following four metrics: spatiotemporal averaged WSS (STA-WSS), spatiotemporal maximum WSS (WSS-max), spatiotemporal averaged minimum WSS (WSS-min), low WSS area (LSA) smaller than 2 Pa (Qin *et al* 2017), and oscillatory shear index (OSI). Specifically, OSI quantifies the change in the direction of the shear stress vector over time; its derivative—relative residence time (RRT)—was also calculated to quantify how long blood remains near the vessel wall.

In addition to the WSS parameters, a vortex analysis was performed following the procedure proposed in previous publications (Sunderland *et al* 2021, Rezaeitaleshmahalleh *et al* 2023c). A previously published vortex analysis method (Sunderland *et al* 2021) was used to extract regions occupied by swirling flow, which have a distinct velocity profile compared to laminar flow regions. The primary parameter, the temporally averaged degree of vortex overlap (DVO), quantifies the movement of vortices within an aneurysm over a cardiac cycle. The volume percentage occupied by the vortex (Vt/V) is also calculated. These vortex-related parameters reveal the temporal stability of blood flow and provide insights into temporal changes in the flow vortex structure.

Furthermore, we implemented the directional velocity-informatics method, which was described by two recent studies (Rezaeitaleshmahalleh *et al* 2023a, 2023c), to qualitatively evaluate the overall patterns of flow direction change. Various features were investigated, including first-order statistics (18 features), gray-level co-occurrence matrix (GLCM, 24 features), gray-level run length matrix (GLRLM, 16 features), and gray-level size zone matrix (GLSZM, 16 features). A brief introduction to the directional velocity informatics method is provided in Supplementary Materials S3.

Comprehensive statistical analyses were conducted to quantitatively evaluate the above-mentioned parameters. These included the Relative Percent Difference (RPD), Linear Regression (LR), Pearson Correlation Coefficient (PCC), and Bland-Altman (BA) analysis (Giavarina, 2015).

Recall that our dataset comprises 112 cases from three anatomical locations in the anterior circulation (ICA, MCA, ACA). A sub-group analysis was conducted to investigate how IA location might affect the results when comparing the Carreau-Yasuda and Newtonian rheology models.

### 2.5. Sensitivity investigation using multiple non-newtonian models

In addition to the CY model, one of the most commonly used rheological models, we also investigated the Casson and Herschel-Bulkley (HB) models using 10 randomly selected cases as part of this sensitivity study. All other CFD simulation procedures mentioned above remained the same, except that a different non-Newtonian blood rheology model was used. The classic Casson model calculates dynamic viscosity based on hematocrit and plasma viscosity. Specifically in this study, the hematocrit was assumed to be 40%, with a plasma viscosity of 1.4mPa⋅s

For the HB model, the shear rate-dependent viscosity η was calculated based on a yield stress threshold $\left(\tau_{0}\right)$, consistency index (k), power-law index (n), and critical shear rate ${\dot{\gamma }}_{c}$, as shown in the following equation:

(5) $$\eta =\frac{\tau_{0}}{\dot{\gamma }}+k{\left(\frac{\dot{\gamma }}{{\dot{\gamma }}_{c}}\right)}^{n-1}$$

where $\tau_{0}=0.01Pa,\;n=0.7,\;k=0.04{kgs}^{n-2}m^{-1}$ and ${\dot{\gamma }}_{c}=0.001{\;s}^{-1}$. (Cho and Kensey, 1991, Wajihah and Sankar, 2023). The CFD results obtained using Casson and HB models were then compared to the result of the Newtonian model.

A plot showing dynamic viscosity in response to the shear rate using three different non-Newtonian rheological models (CY, Casson, and HB) and the Newtonian model can be found in Supplementary Materials (Section S1).

### 2.6. ML-based predictive modeling

In a recent study (Rezaeitaleshmahalleh *et al* 2023c), Jiang *et al* demonstrated that combining geometric parameters with directional velocity informatics can accurately and effectively predict the rupture status of IAs. Notably, the support vector machine (SVM) outperformed other ML methods, including the generalized linear model (GLM), GLM with lasso regularization (GLMNet), and random forest (RF). Therefore, the SVM classifier was utilized in this study to predict the rupture status of the IAs.

Following good practices, a multistep dimensionality reduction approach was employed to eliminate redundant features while retaining the most informative features for prediction. Initially, all features were evaluated using the Wilcoxon rank-sum test, and those with a p-value greater than 0.8 were removed. Because the two CFD models used the same volumetric mesh, a baseline model was constructed using only the morphological parameters. A stepwise feature selection method was then applied to identify the optimal directional velocity-informatics parameters that could improve the performance of the baseline model.

A support vector machine (SVM) model with linear kernels was implemented using R-Studio (Build 764, https://www.r-studio.com/). All 112 cases were split into training and testing datasets in a ratio of 9:1. The model was trained using the train function, and the hyperparameters were tuned using the expandgrid function. A 10-fold cross-validation was performed using the train-control function. To ensure stability and reliability, the entire training and testing process was repeated 100 times, and the average results were used as the outcome and evaluated based on the area under the curve (AUC) and prediction accuracy of the rupturing status. Finally, the SVM models were interpreted using the SHapley Additive exPlanations (SHAP) algorithms (Lundberg and Lee, 2017, Mu *et al* 2023) to understand how each feature contributes to the final predictions.

## 3. Results

### 3.1. Quantitative evaluation of hemodynamics from Newtonian and non-Newtonian model

For the methodology outlined in the Methods and Materials section, ‘patient-specific’ CFD simulations were performed on all 112 cerebral IA cases to calculate hemodynamic parameters. The results for the eight hemodynamic parameters are summarized in table 1. The regression slopes are listed in table 1. The detailed LR and BA plots for each variable are provided in Supplementary Materials S4.

Table 1 compares the hemodynamic parameters obtained from Newtonian and non-Newtonian models. It includes the mean and standard deviation of the relative percent difference (RPD), linear regression slope, Pearson correlation coefficient (PCC), and three key components from the Bland-Altman analysis. The results showed that all WSS-related parameters exhibited relatively high discrepancies (greater than 15%) between the Newtonian and non-Newtonian CY models. Notably, although both OSI and RRT did not differ significantly in terms of the absolute difference, there were approximately 50% (relative) differences, indicating that the OSI and RRT values from the non-Newtonian CY model were larger than those from the Newtonian model.

All hemodynamic parameters exhibited a PCC greater than 0.84 and a slope mostly between 0.80 and 0.99 (see table 1), indicating strong linearity between the two models. However, the Bland-Altman analysis shows that for all WSS-related parameters, there is a large relative difference between the upper and lower limits compared with the average of the means. This indicates that while the methods agree with the trends, the measured values can differ substantially.

Unlike the WSS parameters, the DVO and Vt/V showed excellent agreement between the two models. The high slope and PCC values suggest a strong linear correlation, whereas the RPD and Bland-Altman bias remained low. This high agreement between the vortex-related parameters indicates a strong similarity in the overall flow patterns of the two blood fluid models.

When the entire dataset is divided into sub-groups based on their anatomical locations, as shown in table 2, a more detailed understanding of the shear-thinning effect caused by the CY model becomes evident. Statistical analyses were performed to determine whether the influence of the blood rheology model plays a more significant role in a specific anatomical location. Given that the combination of aneurysm location and flow model forms a two-factor design, an Analysis of Variance (ANOVA) test was used to evaluate both main effects (overall differences between blood rheology models or locations) and interaction effects (whether blood rheology model differences depend on the IA location). However, since the sample size is relatively small (52 MCA, 39 ICA, and 21 ACA) and the Shapiro-Wilk test and Q-Q plot indicated that most hemodynamic parameters do not follow a normal distribution, the traditional two-way ANOVA is unsuitable. Instead, the Aligned Rank Transform (ART) ANOVA was performed using the ARTool package in R Studio (Posit Software, Boston, MA, version 2023.12.1.402). This method adapts traditional ANOVA for non-parametric data that violates assumptions such as normality.

We found that the influences of the blood rheology model varied among different IA locations. For instance, the discrepancy in LSA (area with WSS lower than 2 Pa) is significantly larger (p-value = 0.012) in the ACAs than in the ICAs and MCAs. Also, the difference in Vt/V is significantly higher (p-value = 0.048) in the ICAs than in the ACAs and MCAs. The high variability in the ICAs (i.e., high RPD combined with low PCC) indicates that non-Newtonian effects introduce complex, nonlinear modifications in eddy dynamics. Specifically, the shear-thinning behavior significantly alters recirculation patterns, affecting vortex cores’ formation, stability, and quantification. This effect seemed more pronounced in the ICAs. Of note, most of the ICAs are side-wall IAs, while most ACAs and MCAs are terminal aneurysms.

Furthermore, the discrepancy in WSS-min is significantly larger (p-value = 0.030) in the ACAs and MCAs than in the ICAs.

**Figure 2.**
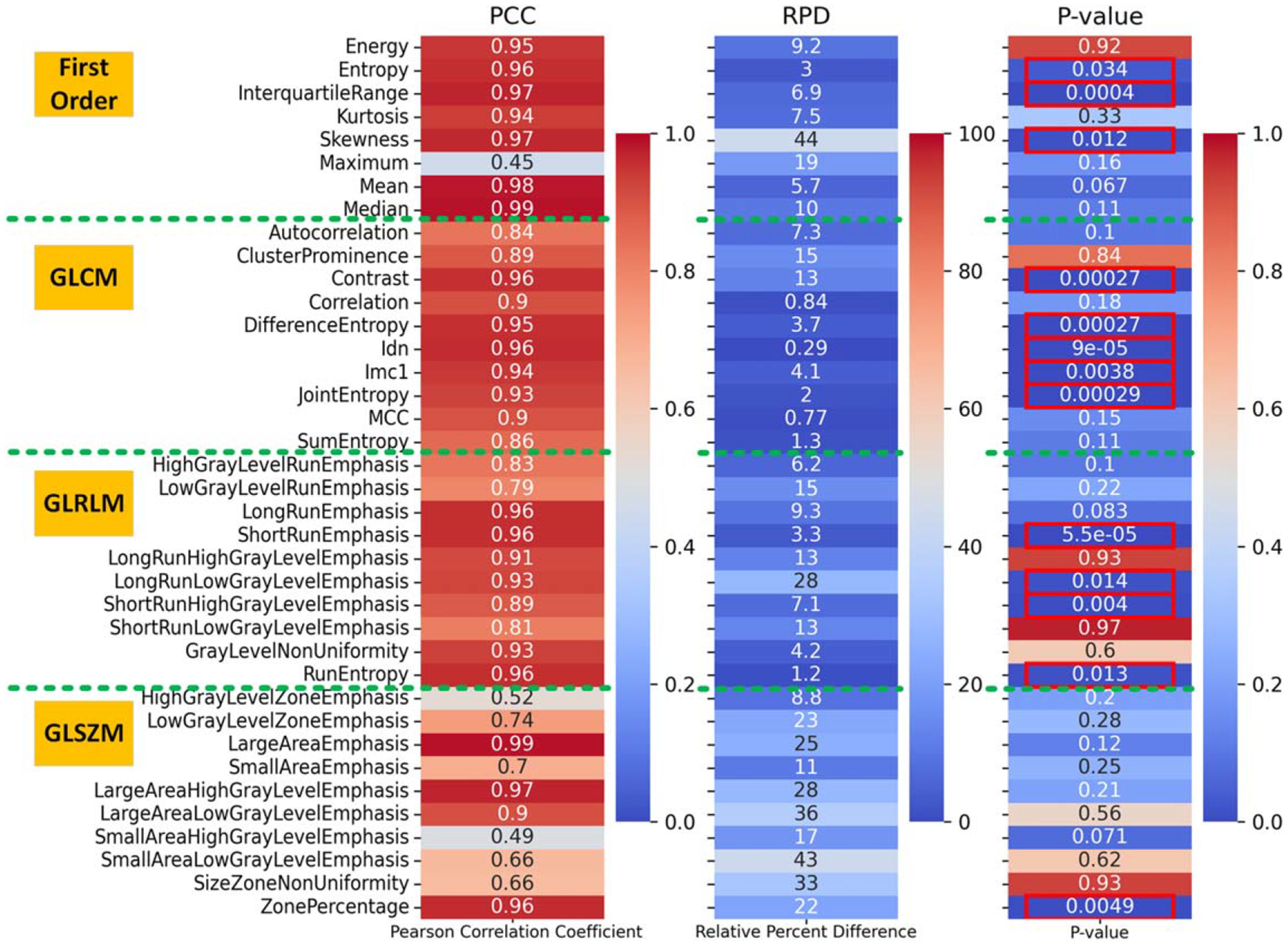
Heat maps depicting PCC, RPD, and P-values for selected velocity informatics parameters, comparing Newtonian and non-Newtonian CY models. P-values, calculated using the paired T-test, highlight statistically significant differences (marked with red boxes) between the two models.

Figure 2 presents the PCC, RPD, and P-values of the velocity informatics parameters to facilitate a comprehensive assessment of overall flow variability. For clarity and brevity, only a subset of the parameters is displayed. A plot of all 74 parameters is shown in Supplementary figure S3. The PCC heat map indicates that most parameters exhibit strong to very strong correlations (from 0.5 to 1.0) between these two different flow models.

Only FirstOrder.Maximum and GLSZM.SmallAreaHighGrayLevelEmphasis showed moderate correlations (approximately 0.4). Moreover, the RPD heat map generally demonstrated a moderate to low discrepancy between the two flow models. Four parameters showed a high RPD (e.g., > 30%). These results align with the DVO findings, indicating that the overall blood pattern exhibited a high level of agreement between the two models (Newtonian model versus CY model). However, the P-values indicate that for many parameters, there are statistically significant differences between the two flow models. The high PCC and low RPD suggest a systematic difference between Newtonian and non-Newtonian CY models.

### 3.2. Visual assessment of overall flow patterns

**Figure 3.**
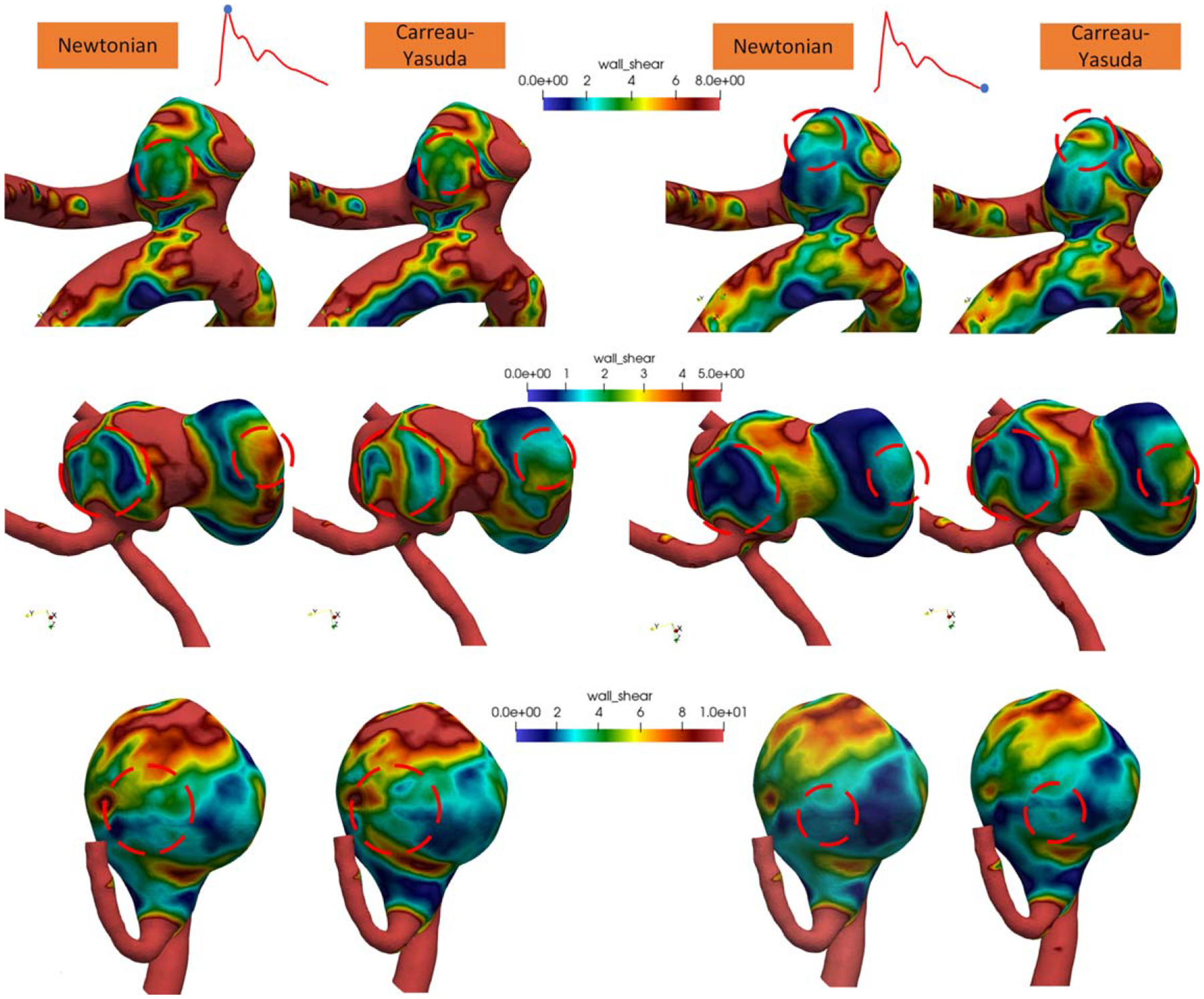
WSS distributions of three cases at systole (left) and diastole (right). WSS values are measured in Pa. Regions with visible differences are highlighted using a red circle.

Gross flow patterns were illustrated using flow velocity streamlines and WSS distributions of the aneurysm structure to better visualize numerical findings. Figure 3 presents the WSS distributions for the three representative cases with varying shapes during the systole and diastole phases. Red indicates the highest WSS values, whereas blue indicates the lowest. The results show discrepancies in the WSS distribution between the Newtonian and non-Newtonian CY models, which is consistent with the high RPD of the WSS parameters shown in table 1. Notably, regions with low WSS (<2 Pa) exhibited higher discrepancies compared to areas with high WSS (e.g., diastolic WSS distribution in Cases 2 and 3).

**Figure 4.**
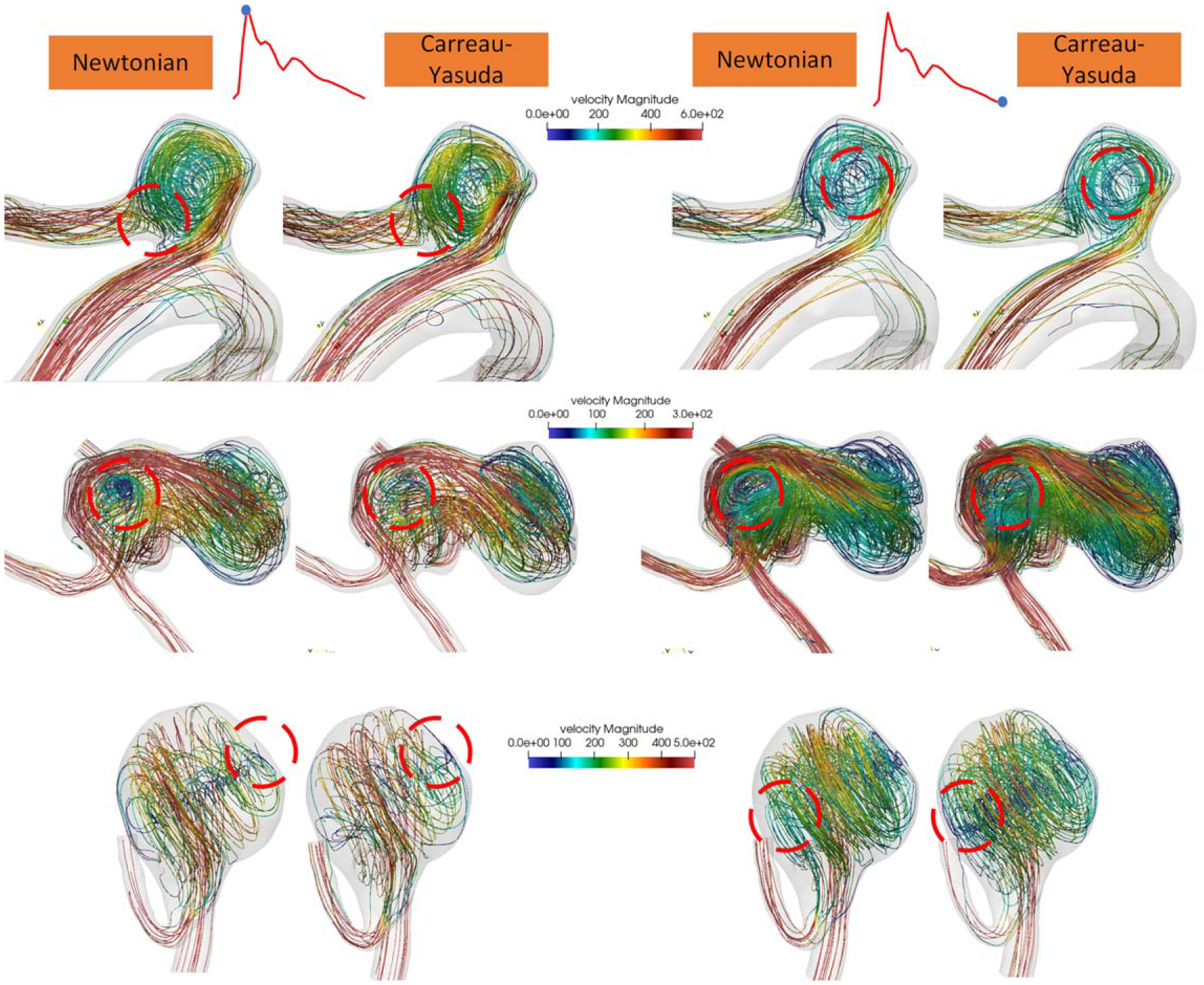
Velocity streamlines of three cases at systole (left) and diastole (right). Velocity values are measured in mm/s. Regions with visible differences are highlighted using red circle

Figure 4 shows the velocity streamlines for the same three representative cases (figure 3) with varying shapes during the systole and diastole phases. Compared to the deviations in the WSS distribution, the flow streamlines exhibited a generally high agreement between the Newtonian and non-Newtonian CY models. Minor variations were observed in the low-velocity regions but did not significantly impact the overall hemodynamic patterns represented by the velocity streamlines. This observation supports the findings of the velocity informatics analysis, which indicated no significant difference in the overall flow patterns between the two models.

### 3.3. Evaluation of ML-based rupture status predictions

Beyond comparing the general hemodynamic results and their derivatives, this study aimed to determine how the results from different CFD models might affect the prediction of IA rupture status. Recall that our dimensionality reduction process begins by generating a baseline model based solely on the morphological parameters. Additionally, all basic hemodynamic parameters showed no improvement compared to the baseline model. The processes are explained in detail elsewhere (Jiang *et al* 2023). Therefore, for the sake of clarity, only velocity informatics parameters are used and discussed in this section.

All 74 velocity informatics parameters were analyzed using the Wilcoxon rank-sum test to identify features with significant differences between the ruptured and unruptured groups. Table 2 highlights the 15 features with the smallest p-values for the Newtonian model (from a total of 46 features with p < 0.05) and non-Newtonian model (from a total of 51 features with p < 0.05). A comprehensive explanation of the velocity informatics parameters is provided in Supplementary Material (section S3). Notably, the two flow models share the same 47 significant features; however, the non-Newtonian CY model has four more features deemed important compared to the Newtonian model. Table 3 presents the four features identified as significant exclusively in the non-Newtonian model.

Table 3 shows the performance of the baseline ML model and additional parameters from different settings that can further improve the baseline ML model. Three second-order velocity informatics parameters —IMC2 from GLCM, difference entropy from GLRLM, and long-run emphasis —were added to the baseline ML model for the Newtonian blood model. These variables improved the AUC from 0.82 to 0.86 and the accuracy of detecting ruptured cases from 45.1% to 60%, with a minimal 2% sacrifice in accuracy for unruptured cases, as reported in a prior publication (Jiang *et al* 2023). First Order. Mean, GLRLM. RunEntropy, and GLCM.DifferenceAverage were added to the baseline ML model for the non-Newtonian CY blood model. This addition further improved the rupture detection rate to 59.75%, which resulted in a drop of less than 1% in the AUC and rupture detection.

Overall, predictive modeling using hemodynamic features derived from Newtonian and non-Newtonian CY simulation models performed comparably, as suggested by table 3.

**Figure 5.**
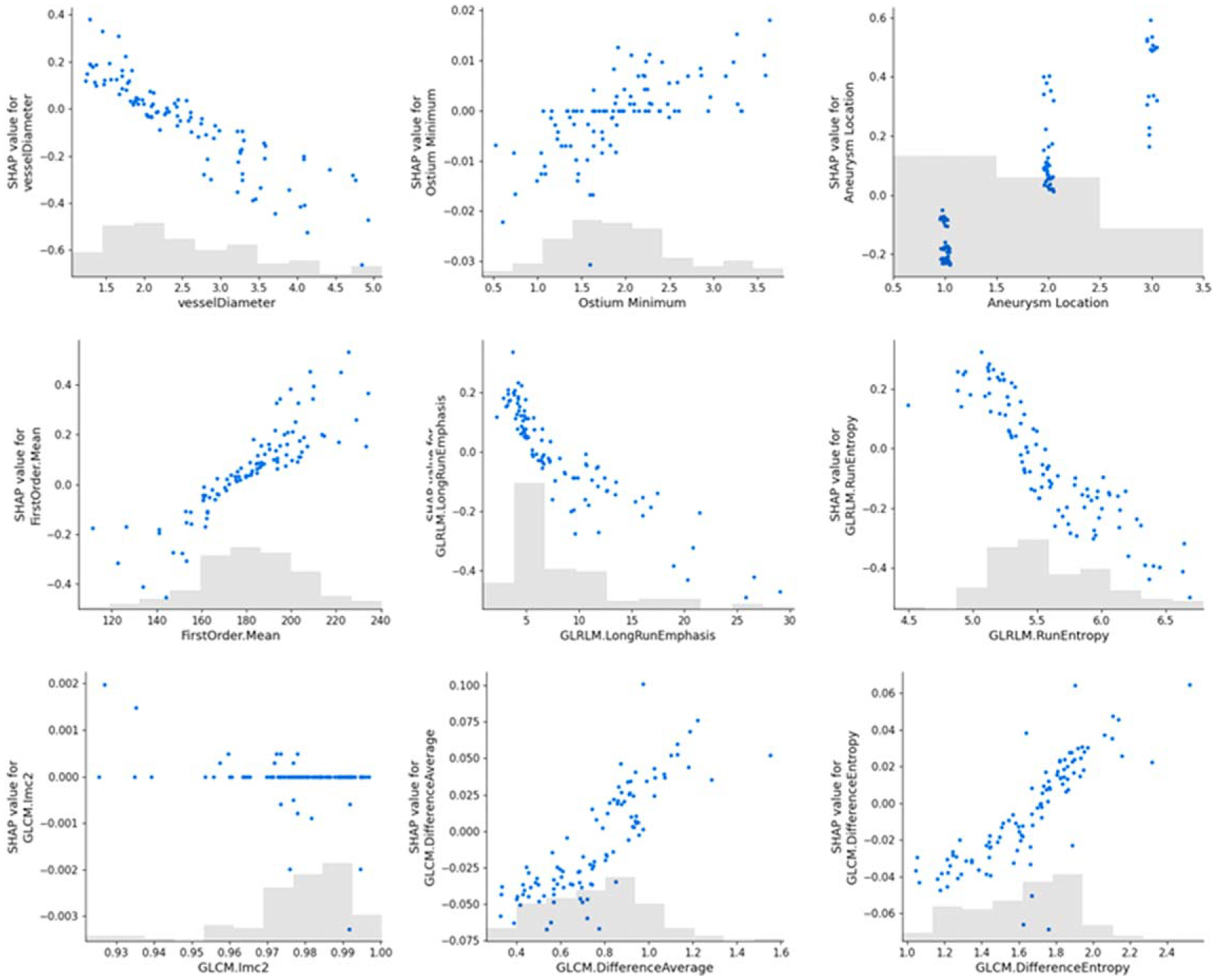
Dependency scatter plots illustrate the impact of a single feature on predictions by the SVM model. Each point represents an individual data point from the dataset. The x-axis indicates the feature’s value, while the y-axis shows the SHAP value of that feature, with positive values indicating ruptured status and negative values indicating unruptured status. This reflects the degree of influence the feature value has on the model’s prediction results. The light gray area at the bottom represents the histogram of the feature value distribution.

To further investigate how each feature contributes to status prediction, we implemented SHAP to generate a scatter plot denoting the importance of the features used in the SVM model. A scatter plot of all features is presented in figure 5. Generally, Aneurysm Location, FirstOrder.Mean, GLCM.DifferenceAverage, and GLCM.DifferenceEntropy are high in terms of feature importance, with higher values contributing more to unruptured predictions. Conversely, (parent) Vessel Diameter, GLRLM.LongRunEmphasis, and GLRLM.RunEntropy are also important, with higher values contributing more to rupture predictions. Ostium Min and GLCM.IMC2 contributed minimally to the predicted results.

### 3.4. An analysis of two additional non-Newtonian models

In ten randomly selected IAs, we also compared results from the Newtonian model to two non-Newtonian Casson and HB models. The results are shown in tables 4 and 5.

The discrepancies between the Newtonian and non-Newtonian Casson models (see table 5) regarding PCC and RPD values are comparable to those between the Newtonian and non-Newtonian CY models (table 1). However, the discrepancies between the Newtonian and non-Newtonian HB models are higher, i.e., lower PCC values (table 1 [average PCC = 0.92] versus table 5 [average PCC = 0.86]). The discrepancies between the Newtonian and non-Newtonian HB models (see table 6) regarding PCC and RPD values are greater, as compared to these shown in table 5.

## 4. Discussion

This study investigated the impact of two different blood rheology models (i.e., Newtonian and non-Newtonian CY models) on CFD-simulated hemodynamic outcomes and subsequent predictive modeling of IA rupture status. Despite the shear-thinning effect of blood, there is currently no consensus on a non-Newtonian blood rheology model for simulating blood flow. Our results demonstrated no significant difference between the Newtonian and non-Newtonian CY models’ overall gross aneurysmal flow pattern and rupture status prediction. This trend seems to hold for the comparisons using two additional non-Newtonian models (Casson and HB). The overall trend in table 1 is consistent with the results in tables 5 and 6. Discrepancies were observed in the spatiotemporally averaged WSS and the distribution of the WSS pattern, particularly showing higher discrepancies in the low WSS regions during the diastolic period. These findings are consistent with those of previous studies (Bernsdorf and Wang 2009, Hippelheuser *et al* 2014, Oliveira *et al* 2021, Hosseini *et al* 2022, Yi *et al* 2022). Moreover, the non-Newtonian model showed lower peak WSS and higher OSI values (Huang *et al* 2018, Xu *et al* 2018).

### 4.1. Hemodynamics and velocity informatics differences between different flow models

The parameters directly measured from the CFD simulations, as summarized in table 1, showed an average Pearson correlation coefficient (PCC) of 0.92 across the six hemodynamic variables, indicating a high degree of correlation between the two models. Additionally, an average slope of 0.89 demonstrates strong linearity. Despite these indicators of correlation and linearity, the Bland-Altman analysis and relative percent difference (RPD) revealed considerable variability in the differences between the methods. However, this variability in the WSS distribution did not overshadow the highly consistent overall flow patterns quantified by vortex analysis and velocity informatics, as shown in figures 3 and 4. It is worth noting that the non-Newtonian CY and Casson models show a slightly higher OSI but a lower RRT. This suggests that the non-Newtonian CY and Casson simulations produce more substantial shear stresses near the wall and somewhat more oscillatory flow. In contrast, the Newtonian model predicts lower shear stress near the wall and longer residence times. The non-Newtonian model’s higher OSI and lower RRT may better reflect the true hemodynamic stress on the aneurysm wall.

A clear trend emerges: the majority of discrepancies occur in regions characterized by low velocity and low shear, which is consistent with findings from previous studies (Bernsdorf and Wang 2009, Hippelheuser *et al* 2014, Oliveira *et al* 2021, Hosseini *et al* 2022, Yi *et al* 2022). These discrepancies are reflected in the relatively high RPD values for the WSS parameters in table 1 and the statistically significant differences in several velocity informatics parameters shown in figure 2. Combined with the high linearity observed across most parameters, it is evident that the differences between the two models are minor but systematic.

Some velocity informatics parameters highlight the differences between the Newtonian and non-Newtonian CY models. For example, **FirstOrder.Skewness** (RPD: 44.19%, P-value: 0.0122), which quantifies the asymmetry of the velocity distribution relative to the mean, exhibits significant differences between the two models. The high RPD and low P-value indicate that non-Newtonian effects substantially reduce the asymmetry in velocity direction distributions, likely smoothing abrupt directional changes in high-shear regions. This reduction was confirmed by the lower average and standard deviation of the non-Newtonian CY model. As shown in figure 4, the Newtonian streamline demonstrates more flow disturbance in the low-velocity regions than in the low-velocity regions in the smoother non-Newtonian CY flow.

Another critical parameter is **GLSZM.ZonePercentage** (RPD: 21.57%, P-value: 0.0049), which measures the proportion of the aneurysm region dominated by specific flow directions. Significant differences in this parameter suggest that shear-thinning effects redistribute flow directions and alter the low-shear regions (i.e., flow stagnation regions). This redistribution could have implications for thrombus formation, as thrombosis is typically initiated in regions with low shear.

Similarly, **GLRLM.LongRunLowGrayLevelEmphasis** (RPD: 27.70%, P-value: 0.0143) highlighted areas where inflow jet directions remained consistent. Recall that low gray values in the directional velocity is aligned with the inflow jet direction entering an IA. That also explained why the gross aneurysmal flow patterns remained similar.

Finally, **FirstOrder.Entropy** (RPD: 3.01%, P-value: 0.0342), which quantifies the randomness of the flow directions, also shows significant differences between the models. Lower entropy values correspond to more organized flows. The low RPD, combined with the significant P-value, suggests that non-Newtonian effects introduce small but meaningful reductions in the flow randomness. The reduced entropy in the non-Newtonian CY model indicated a shift toward more organized flow patterns.

In summary, metrics such as **LongRunLowGrayLevelEmphasis** and **ZonePercentage** demonstrated increased directional persistence and redistribution of flow zones, suggesting that non-Newtonian CY models more accurately capture conditions that promote flow stagnation, a precursor to thrombus formation. These effects are particularly pronounced in the low-shear regions within the aneurysm sac. **Skewness** and **Entropy** metrics further support this, as reduced skewness and entropy in non-Newtonian models indicate a more organized slow flow.

It is worth noting that additional plots in Supplementarty Materials (Section S6) show that the overall trend of the differences among selected velocity informaitcs metrics between the Newtonian and Casson or HB models is consistent with what is displayed in figure 2 and discussed above.

### 4.2. Variation in the ML models

These observations align with the findings in table 4, which demonstrate that both Newtonian and non-Newtonian blood rheology models can effectively predict the rupture status of IAs with high accuracy (AUC ⩾ 0.85). However, minor local differences between the two models resulted in variations in the parameters utilized by the support vector machine (SVM) model.

Velocity informatics analysis shows greater potential for rupture status prediction compared to traditional hemodynamic parameters, which do not enhance the predictive capability of the baseline model. Notably, the four parameters listed in table 3, which distinguish ruptured from unruptured aneurysms exclusively in the non-Newtonian model, reveal their impact on flow distribution. For example, GLCM.JointAverage and GLCM.SumAverage quantify the spatial relationships and co-occurrence probabilities of pixel intensities (representing the velocity directions). Non-Newtonian models capture more nuanced flow dynamics and yield measurable changes in these spatial relationships, particularly in ruptured aneurysms.

Additionally, FirstOrder.Mean and FirstOrder. Range, which summarize the pixel intensity distributions, highlight how non-Newtonian flow models create more dynamic and heterogeneous velocity profiles, leading to significant metric differences.

These significant variations underscore the superior potential of the non-Newtonian CY model to capture intricate flow patterns. However, our predictive modeling of IA rupture status did not find using non-Newtonian CY blood model yielded improved results (see table 4). In the future, we will further explore novel hemodynamic metrics associated with rupture-prone IAs.

### 4.3. Limitations and future outlook

4D magnetic resonance angiography (MRA) can obtain ‘patient-specific’ flow information and be used to establish more realistic boundary conditions. However, 4D MRA is not routinely used for the standard care of patients with brain aneurysms. If ‘patient-specific’ CFD simulations can be verified at a large scale (~100) using *in vivo* 4D MRA data, the confidence of computed aneurysmal hemodynamics will be improved. Moreover, ‘patient-specific’ boundary conditions (e.g., flow waveforms or flow divisions) will be available for studies like ours. We consider this a limitation of our study.

In this study, only the classical SVM classifier is used. In the future, particularly when a larger database becomes available, we are interested in using spatially varying wall shear stress (WSS) in conjunction with deep learning-based classifiers (e.g., graph neural network) to improve the characterization of IAs’ rupture status.

Another limitation is that only the non-Newtonian CY model was used to study how the non-Newtonian effects may influence the characterization of IAs’ rupture status. A similar trend persisted when we compared three non-Newtonian models to Newtonian ones. However, such comparisons were only applied to 10 randomly selected cases.

Because *in vivo* flow rate measurements are often not available for a large cohort of patients, computational hemodynamic studies largely adopted one of two outlet boundary conditions: (1) variants of the Murray’s law (Xiang *et al* 2011, Chnafa *et al* 2018, Detmer *et al* 2018a, 2018b) and (2) zero-pressure outlet boundary conditions (Xu *et al* 2018, Wan *et al* 2019, Chen *et al* 2022). We compared the zero-pressure outlet boundary condition used in the study with a variant of Murray’s law proposed by Chnafa *et al* (Chnafa *et al* 2018). We found that the change of outlet boundary conditions impacted the eight hemodynamic metrics (up to RPD of 70%). Our results are consistent with an earlier study by Korte *et al* (Korte *et al* 2023). These results are included in Supplementary Materials (section 5) for completeness.

Our primary goal is to investigate the sensitivity of ML-based predictions induced by blood rheology models. Now we know that boundary conditions may play an even larger role regarding their impacts on ML-based rupture status prediction. The combined effects should be understood; that will be left as our future work.

To translate computational hemodynamics into the clinical workflow for managing cerebrovascular diseases, the standardization of CFD simulations is important. The outlet boundary condition proposed by Chnafa *et al* is physiologically sound and easy to implement. The future standardization should consider adopting this outlet boundary. Second, when we advocate the usefulness of hemodynamic markers for clinical use, we should also consider hemodynamic metrics less sensitive to the blood rheology models. For instance, as shown in tables 1, 5, and 6, OSI values are small and are known to have relatively high fluctuations. In contrast, velocity informatics parameters appeared more robust concerning the change of blood rheology models. The sensitivity of velocity informatics when two other non-Newtonian Casson and HB models can be found in Supplementary Materials (section S6). Thus, we think the potential of velocity informatics metrics should be further explored.

## 5. Conclusions

This study indicates that the flow patterns in Newtonian and non-Newtonian blood models exhibit no significant differences. However, investigations into WSS and its derivatives should prioritize non-Newtonian models to approximate the *in vivo* blood flow properties more accurately. Our velocity informatics analysis introduces a novel perspective for evaluating velocity distributions, revealing more insights than traditional methods and demonstrating the potential for accurately predicting aneurysm rupture status. Interestingly, our ML models built on Newtonian and non-Newtonian CFD simulations did not show differences regarding the accuracy of predicting IA’s rupture status.

## Supplementary Material

Supplementary_materials

## Figures and Tables

**Table 1.** Comparison of eight hemodynamic parameters simulated using Newtonian and non-Newtonian CY models. RPD is presented as (Mean ± SD). The slope represents the relationship between the Newtonian (x-axis) and non-Newtonian (y-axis) models. Upper limit (up-lim) and lower limit (low-lim) represent the 95% confidence interval from the BA analysis.

Parameter	Mean value ± SD		RPD (%)	Slope	PCC	Bias	Up-lim	Low-lim
	Newtonian	CY						
STA-WSS (Pa)	6.84 ± 6.12	5.97 ± 5.53	17.20 ± 18.35	0.89	0.98	−0.87	1.55	−3.29
WSS-max (Pa)	55.83 ± 51.96	46.99 ± 45.35	18.43 ± 24.53	0.84	0.90	−8.83	19.94	−37.6
WSS-min (Pa)	0.46 ± 0.84	0.42 ± 0.75	32.03 ± 29.68	0.80	0.96	−0.04	0.68	−0.76
LSA (%)	37.07 ± 26.31	41.36 ± 27.57	21.48 ± 25.67	0.98	0.93	4.29	23.63	−15.04
OSI	0.02 ± 0.01	0.03 ± 0.02	47.95 ± 23.61	0.96	0.85	0.01	0.03	−0.01
RRT	1.04 ± 1.78	0.62 ± 0.96	52.33 ± 21.85	1.56	0.84	−0.42	2.57	−1.72
DVO	0.70 ± 0.18	0.74 ± 0.14	9.83 ± 9.89	0.84	0.90	0.04	0.19	−0.11
Vt/V	0.25 ± 0.10	0.25 ± 0.10	9.90 ± 13.37	0.99	0.97	0.00	0.05	−0.05

**Table 2.** Sub-group study based on the anatomical location of the aneurysm. RPD is presented as (Mean ± SD). The slope represents the relationship between the Newtonian (x-axis) and non-Newtonian (y-axis) models.

Parameter	ACA			MCA			ICA		
	RPD (%)	Slope	PCC	RPD (%)	Slope	PCC	RPD (%)	Slope	PCC
STA-WSS (Pa)	15.52 ± 9.71	0.83	0.99	17.75 ± 24.47	0.97	0.97	17.29 ± 12.00	0.88	0.99
WSS-max (Pa)	19.22 ± 27.64	0.83	0.96	21.29 ± 28.84	0.86	0.97	14.39 ± 15.26	0.81	0.95
WSS-min (Pa)	31.64 ± 25.75	0.62	0.92	32.72 ± 34.66	0.94	0.92	29.21 ± 22.55	0.73	0.95
LSA (%)	28.29 ± 42.73	1.11	0.96	16.53 ± 17.75	1.03	0.95	14.38 ± 21.93	0.90	0.90
OSI	32.00 ± 21.26	1.22	0.85	39.73 ± 22.49	0.88	0.87	37.47 ± 24.83	1.20	0.96
RRT	46.64 ± 16.13	0.73	0.87	55.45 ± 32.05	0.92	0.98	53.88 ± 21.03	0.72	0.95
DVO	9.87 ± 10.08	0.92	0.92	9.48 ± 9.16	0.83	0.89	10.43 ± 10.99	0.79	0.91
Vt/V	7.15 ± 6.08	0.95	0.97	10.74 ± 13.14	0.99	0.96	23.84 ± 27.52	0.78	0.63

**Table 3.** Important velocity informatics features selected by the Wilcoxon rank-sum test. A p-value < 0.05 indicates significant differences between ruptured and unruptured IAs. The last two columns show each feature’s value (Mean ± standard deviation) in different aneurysm groups.

Flow model	Feature	P-value	Ruptured IAs	Unruptured IAs
Newtonian	GLCM.JointAverage	0.131	7.98 ± 0.83	7.69 ± 0.93
	GLCM.SumAverage	0.131	15.95 ± 1.66	15.38 ± 1.87
	FirstOrder.Mean	0.162	182.66 ± 20.16	176.52 ± 22.09
	FirstOrder.Range	0.397	234.29 ± 23.35	230.38 ± 23.69
Non-Newtonian	GLCM.JointAverage	0.013	8.18 ± 0.94	7.70 ± 0.99
	GLCM.SumAverage	0.013	16.36 ± 1.89	15.41 ± 1.97
	FirstOrder.Mean	0.024	186.70 ± 21.76	176.45 ± 23.59
	FirstOrder.Range	0.035	236.92 ± 19.94	228.22 ± 24.43

**Table 4.** The best performance of the SVM model was achieved using the baseline model and two blood fluid models (i.e., Newtonian and non-Newtonian CY models). ${\text{NRV}}_{2}$ is derived from the Voronoi Diagram Curve.

SVM model	AUC	Ruptured accuracy	Unruptured accuracy
Baseline model (aneurysm location, parent vessel diameter, Ostium min, ${\text{NRV}}_{2}$)	0.82	45.1%	90.1%
Newtonian model (Additional velocity-informatics parameters: GLCM.IMC2, GLCM.DifferenceEntropy, GLRLM.LongRunEmphasis)	0.86	60.00%	88.10%
non-Newtonian model (Additional velocity-informatics parameters: FirstOrder. Mean, GLRLM.RunEntropy, GLCM.DifferenceAverage)	0.85	59.75%	89.33%

**Table 5.** Comparison of eight hemodynamic parameters simulated using Newtonian and Casson models. RPD is presented as (Mean ± SD). The slope represents the relationship between the Newtonian (x-axis) and non-Newtonian (y-axis) models. Upper limit (up-lim) and lower limit (low-lim) represent the 95% confidence interval from the BA analysis.

Parameter	RPD (%)	Slope	PCC	Bias	Up-lim	Low-lim
STA-WSS (Pa)	20.47 ± 13.32	0.76	0.89	0.87	2.76	−1.02
WSS-max (Pa)	37.14 ± 36.45	0.61	0.97	9.58	35.76	−16.60
WSS-min (Pa)	36.48 ± 25.53	0.98	0.84	0.05	0.34	−0.25
LSA (%)	24.36 ± 18.62	1.02	0.91	−1.20	13.81	−16.21
OSI	16.38 ± 15.69	0.94	0.94	0.00	0.01	−0.01
RRT	60.49 ± 30.97	0.95	0.91	−0.23	0.04	−0.51
DVO	8.53 ± 6.37	0.84	0.82	−0.03	0.11	−0.17
Vt/V	15.55 ± 9.08	1.03	0.99	−0.03	0.00	−0.07

**Table 6.** Comparison of eight hemodynamic parameters simulated using Newtonian and non-Netowian HB models. RPD is presented as (Mean ± SD). The slope represents the relationship between the Newtonian (x-axis) and non-Newtonian (y-axis) models. Upper limit (up-lim) and lower limit (low-lim) represent the 95% confidence interval from the BA analysis.

Parameter	RPD (%)	Slope	PCC	Bias	Up-lim	Low-lim
STA-WSS (Pa)	31.97 ± 13.93	0.65	0.91	1.64	3.54	−0.26
WSS-max (Pa)	43.90 ± 23.54	0.55	0.95	11.21	41.59	−19.18
WSS-min (Pa)	41.36 ± 34.69	0.99	0.83	0.04	0.35	−0.26
LSA (%)	36.38 ± 28.22	1.48	0.80	−8.59	19.73	−36.91
OSI	36.08 ± 29.93	0.40	0.77	0.01	0.03	−0.02
RRT	59.91 ± 27.60	0.99	0.94	−0.22	−0.01	−0.77
DVO	15.65 ± 16.39	1.05	0.75	0.09	0.30	−0.12
Vt/V	42.31 ± 29.99	0.89	0.94	0.07	0.13	0.01

## Data Availability

The data cannot be made publicly available upon publication because no suitable repository exists for hosting data in this field of study. The data that support the findings of this study are available upon reasonable request from the authors.
