# Supplementary material for: Investigating the role of blood models in predicting rupture status of intracranial aneurysms
Source: Biomed Phys Eng Express. Author manuscript; Available in PMC 2025 Dec 29. (PMC12747726; doi:10.1088/2057-1976/adcc34)
Supplement: Supplementary_materials [file NIHMS2073724-supplement-Supplementary_materials.pdf]

Supplementary Materials

S1 – A dynamic viscosity plot of four different rheological models

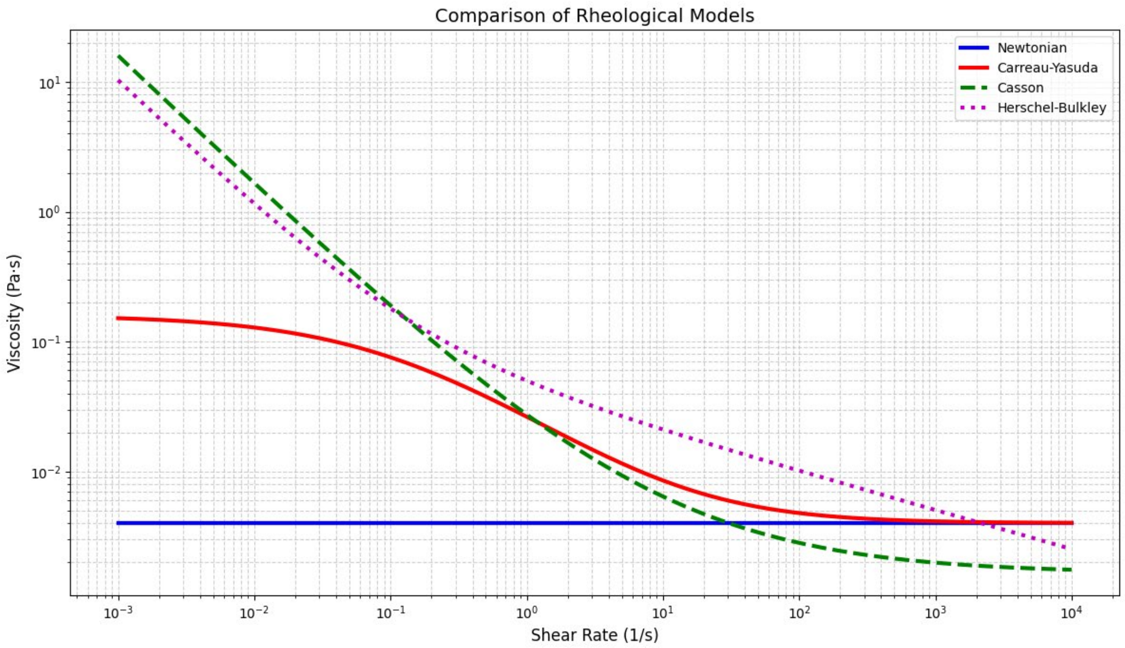

Figure S1: Visualization of dynamic viscosity as a function of the shear rate using this study's four different rheological models.

S2 - Morphological analysis and Voronoi diagram curve (VDC)

As discussed in Methods and Materials Section of the manuscript, we first isolated the auemruysm sac for each intracranial aneurysm (IA), as shown in Fig. S1. Then, the following morphological parameters were calculated (Table S1) and used to produce a baseline model for machine learning (ML) predictions. The ML-based predictive modeling processes are detailed in the manuscript and our previous publication (Jiang *et al.*, 2023).

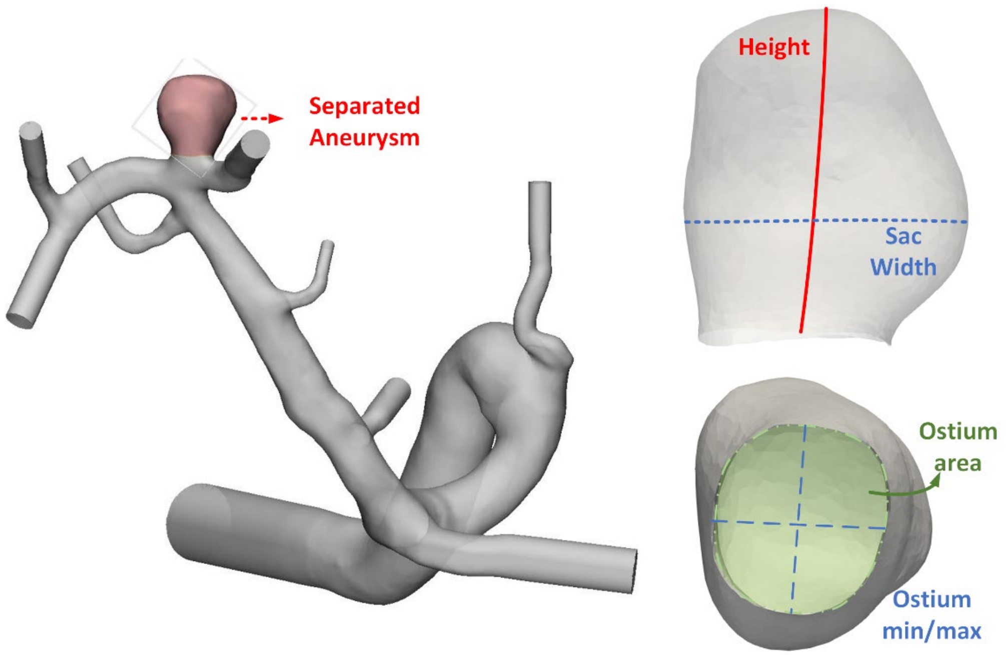

Figure S2. An illustration of the geometric characteristics of typical IAs is provided. (a) shows an isolated IA sac from the parent vessel (highlighted in red). In (b), the solid red lines represent the centerlines generated using the Voronoi diagram (Piccinelli *et al.*, 2009), where the height of the IA is determined by the length of the centerline within the aneurysm region. The blue dase line indicates the maximum width of the IA. In (c), the green circle represents a 2D cutting plane used to measure the minimum and maximum ostium diameters, and the green surface area on the cutting plane indicates the ostium area

Table S1. A summary of morphological parameters used in this study

Parameter	Description
Aneurysm height	Height of aneurysm
Aneurysm volume	Volume of aneurysm
Aneurysm area	Surface area of the aneurysm
Sac width	Maximum width of the aneurysm sac
Vessel diameter	Diameter of the parental vessel connected to the aneurysm
Size ratio width	The size ratio between aneurysm width and parental artery diameter
Ostium minimum	The minimal ostium diameter
Ostium maximum	The maximal ostium diameter
Ostium area	Area of the ostium

Another analysis derived from the isolated IA sac is the VDC (Piccinelli *et al.*, 2009). As shown in Figure S3, an aneurysm volume can be broken down into numerous overlapping spheres of varying sizes, known as maximal inscribed spheres (MIS). Small MISs (illustrated by the blue spheres in Figure S3A) often represent minor protrusions. Removing a fraction of these MISs, starting from the smallest to the largest, gradually reduces the "summed" aneurysm volume, as depicted in Figure S3. This reduction curve is referred to as the Voronoi diagram characteristic (VDC) evolution curve.

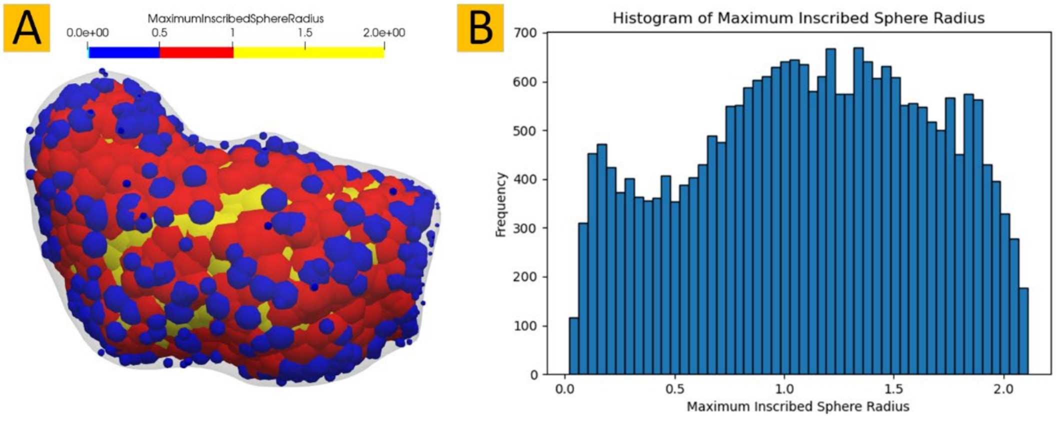

Figure S3: (A) An illustration example showing an aneurysm (the transparent white dome) can be summed together by many overlapping spheres of varying size and (B) A histogram showing a radii distribution of those overlapping spheres ranging from 0.02 to 2.2 mm. In (A), the aneurysm is shown by a transparent white surface, while blue, red, and yellow colors represent spheres of different sizes.

Initially, when small MISs are removed from the "volume summation" process, the decrease in the "summed" aneurysm volume is minimal. However, as larger spheres (closer to the largest MIS) are removed, the "summed" aneurysm volume significantly decreases. Since different aneurysms have varying distributions of MISs (as seen in Figure S3B), the shape of this VDC evolution curve will differ.

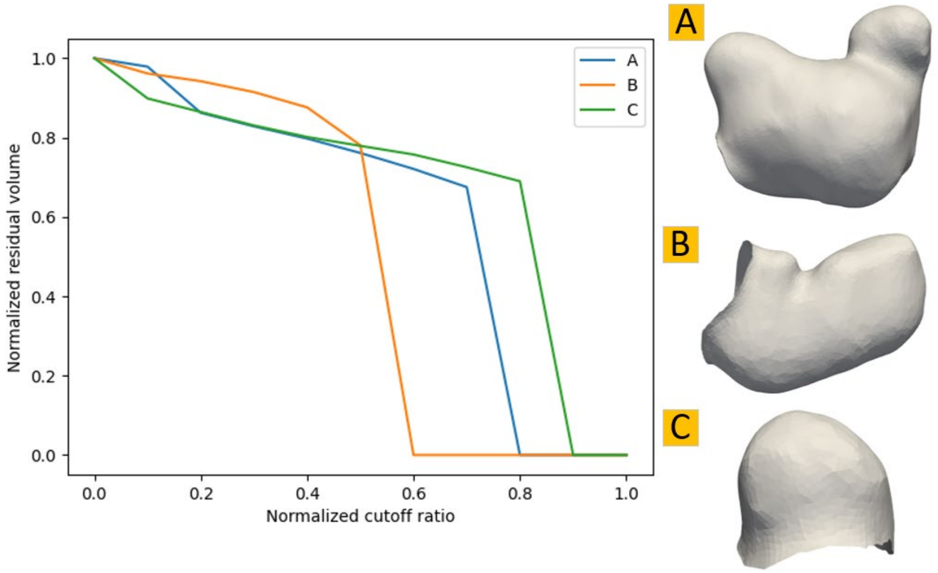

Figure S4: Voronoi diagram curve (VDC) curves for three selected intracranial aneurysms

In Figure S4, for a more spherical aneurysm (Aneurysm A), the "summed" volume reduction decreases slowly as the cutoff ratio increases. In contrast, for two less spherical aneurysms (Aneurysms B and C), the reduction in residual aneurysm volume is much quicker. This observation supports our use of the VDC curve to enhance the differentiation of intracranial aneurysms (IAs). To achieve this, we define NRVx as the normalized residual volume when the cutoff ratio is x/10. Consequently, our machine learning methods can utilize a series of geometrical parameters, NRVx, for IA characterization.

The average value of all morphological parameters and VDC is displayed in Table S2.

Table S2. IA morphological and VDC parameters used for support vector machine (SVM). Parameters are listed as mean ± one standard deviation. Features marked with an asterisk indicate significant differences between ruptured and unruptured IAs: non-parametric Wilcoxon rank-sum test, $p < 0.05$. Only variables with p-values less than 0.35 are displayed in the table.

Paramete	Ruptured IAs	Unruptured IAs	p-value
Aneurysm height (mm)	6.60 ± 2.67	7.80 ± 4.00	0.166
Aneurysm volume (mm^3)	149.92 ± 194.775	280.73 ± 355.14	0.02
Aneurysm area (mm^2)	114.41 ± 97.32	176.04 ± 154.06	0.007
Sac width (mm)	6.54 ± 3.07	7.79 ± 3.03	0.011
Vessel diameter (mm)	2.09 ± 0.76	2.77 ± 0.86	<0.001
Size ratio width	3.51 ± 1.98	2.95 ± 1.39	0.186
Ostium minimum (mm)	1.63 ± 0.58	2.15 ± 0.68	<0.001
Ostium maximum (mm)	2.52 ± 0.96	3.10 ± 1.04	0.001
Ostium area (mm^2)	14.58 ± 11.12	25.29 ± 20.33	<0.001
NRV_2	0.94 ± 0.03	0.95 ± 0.02	0.013
NRV_3	0.90 ± 0.05	0.92 ± 0.05	0.018
NRV_4	0.84 ± 0.09	0.88 ± 0.07	0.013
NRV_5	0.76 ± 0.20	0.84 ± 0.09	0.007
NRV_6	0.68 ± 0.25	0.79 ± 0.17	0.004
NRV_7	0.53 ± 0.34	0.67 ± 0.29	0.005
NRV_8	0.38 ± 0.36	0.53 ± 0.38	0.008
NRV_9	0.17 ± 0.31	0.36 ± 0.40	0.007
NRV_{10}	0.07 ± 0.23	0.25 ± 0.37	0.005

S3 - Velocity informatics

This study employs the velocity-informatics technique to quantify blood flow characteristics using spatial patterns, as proposed in the previous publication (Jiang *et al.*, 2023).

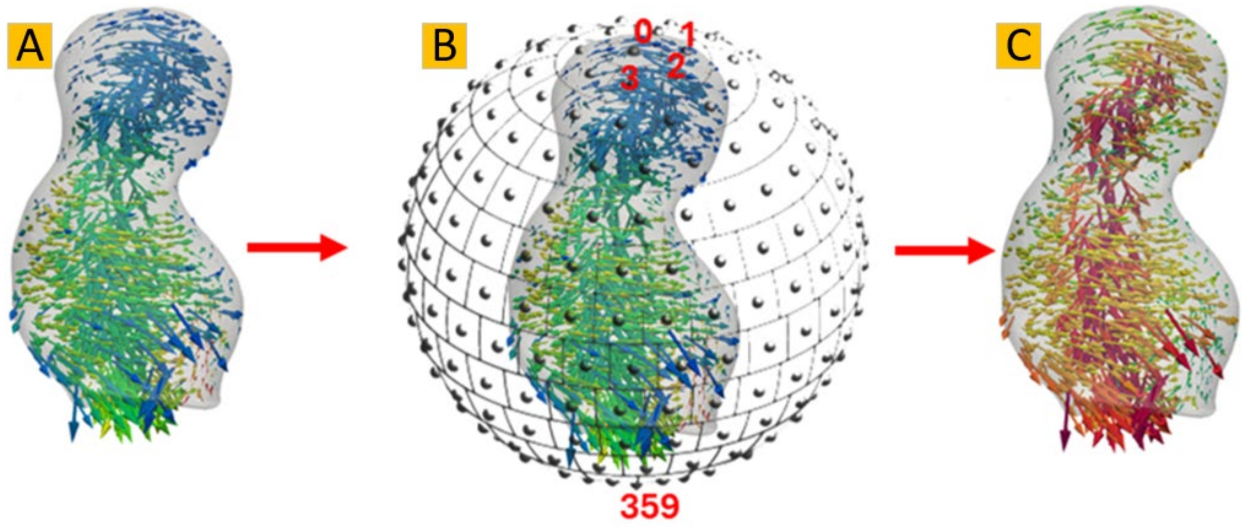

Figure S5: A graphical illustration of the procedures involved for calculating Directional velocity informatics: (a) isolating velocity vectors within IA, (b) utilizing Leopardi's method for defining velocity directions (each partition on the unit sphere corresponds to one unique direction, and (c) computed angular directions for every velocity vector. Recall that all velocity vectors were converted to a rectilinear grid; thus, this process yielded 3D DVelocity images.

As illustrated in Figure S5, starting with a three-dimensional (3D) CFD-simulated velocity field at the peak systole phase in and around an intracranial aneurysm (IA), a previously published IA segmentation algorithm (Jiang and Strother, 2013) is first used to isolate the vector velocity field within the aneurysm dome. All identified vector velocity values in the unstructured grid are then resampled (via interpolation) onto a uniform computing grid with a voxel size of $0.2 \times 0.2 \times 0.2$ mm^3. In the second step (Figure S5B), the direction of each velocity vector is mapped onto an equally partitioned unit sphere divided into 360 sections. Specifically, if a velocity vector's direction aligns best with a particular partition zone vector, it is associated with that specific partition zone. Consequently, each velocity vector in the uniform grid is linked to one of the 360 partitions on the unit sphere (Figure S5B). These velocity associations are treated as a three-dimensional 8-bit image, referred to as a directional velocity field/image (Figure S5C).

Once the 3D directional velocity image was obtained, the open-source Pyradiomics package (van Griethuysen *et al.*, 2017) was used to calculate velocity-informatics parameters in the third step. It is important to note that the Pyradiomics package (https://pyradiomics.readthedocs.io/en/latest/) was applied to a directional velocity image rather than a radiographic image. The descriptions below are similar to those provided in PyRadiomics documentation and are provided below for completeness.

S3.1 - First-Order Statistics

First-order statistical features represent the distribution of individual voxel values without considering their relationships with neighboring voxels. These histogram-based properties include the mean, median, maximum, and minimum values of voxel intensities in the image-like hemodynamic data and measures of asymmetry, flatness, uniformity, and entropy.

S3.2 - Second-Order Statistics:

In contrast, second-order statistical features pertain to the interrelationships between neighboring voxel intensities and their spatial arrangements. Several methods have been reported to quantify these interrelationships, including the Gray Level Co-occurrence Matrix (GLCM), the Gray Level Run Length Matrix (GLRLM), and the Gray Level Size Zone Matrix (GLSZM). Parameters derived from GLCM, GLRLM, and GLSZM are summarized below.

The Gray Level Co-occurrence Matrix (GLCM) quantifies the spatial relationship between pairs of connected voxels based on their intensity values. It is denoted as $P(i, j \mid \delta, \theta)$, where δ represents the distance between the voxels and θ represents the angle of their relative orientation. For 2D images, θ takes on one of four discrete values, increasing to 13 discrete values for 3D images.

Mathematically, the GLCM of an image (or image-like data) with $Nx \times Ny$ dimensions and Ng different intensity levels is computed using the following equation:

$$GLCM_{\delta}^{\theta}(i,j) = \left|\left\{\left((r,s),(t,v)\right): I(r,s) = i, I(t,v) = j\right\}\right| \forall\, i,j \in \{1,2,3,\dots,N_g\} \quad (1)$$

Where (t, v) is defined as:

$$(t,v) = \begin{cases} (r+\delta, s) & if\ \theta = 0° \\ (r+\delta, s+\delta) & if\ \theta = 45° \\ (r, s+\delta) & if\ \theta = 90° \\ (r-\delta, s-\delta) & if\ \theta = 135° \end{cases}$$

and |. | represents the number of components in a set.

Table S3: A summary of GLCM variable

Paramete	Description
Autocorrelation	Quantifies the magnitude of fineness and coarseness of an image texture and can be estimated as: $Autocorrelation = \sum_{i=1}^{N_g}\sum_{j=1}^{N_g} p(i,j)i$
Cluster Shade	Quantifies the level of skewness and uniformity of GLCM, and its higher value implies more significant asymmetry around the average value. $Cluster\ Shade = \sum_{i=1}^{N_g}\sum_{j=1}^{N_g}(i+j-\mu_x-\mu_y)^3\, p(i,j)$
Cluster Tendency	Measures sets of voxels with an identical intensity value. $Cluster\ Tendency = \sum_{i=1}^{N_g}\sum_{j=1}^{N_g}(i+j-\mu_x-\mu_y)^2\, p(i,j)$
Idm	Quantifies the local homogeneity of an image. It assesses how similar intensity values are within neighboring pixel pairs, with higher values indicating greater homogeneity. $Idm = \sum_{i=1}^{N_g}\sum_{j=1}^{N_g}\frac{P_{x-y}(\kappa)}{1+\kappa^2}$
Joint Average	Compute mean intensity level i distribution. $Joint\ Average = \mu_x = \sum_{i=1}^{N_g}\sum_{i=1}^{N_g} p(i,j)i$
Joint Entropy	Quantifies the range of randomness in adjacent intensity values. $Joint\ Average = \mu_x = \sum_{i=1}^{N_g}\sum_{j=1}^{N_g} p(i,j)i$
Joint Energy	Estimates the homogeneity of a pattern in the image. $Joint\ Energy = \mu_x = \sum_{i=1}^{N_g}\sum_{j=1}^{N_g}(p(i,j))^2$

Maximum Probability	Quantifies the number of the most common set of adjacent intensity levels. $Maximum\ Probability = \max(p(i,j))$
Sum Average	Estimates the association between the occurrences of connected pixels with lower and higher intensity levels. $Sum\ Average = \sum_{\kappa=w}^{2N_g} P_{x+y}(\kappa)\kappa\ ,\ where\ P_{x+y}(\kappa) = \sum_{i=1}^{N_g}\sum_{j=1}^{N_g} p(i,j)$ and $i+j=\kappa\ ,\kappa = 2,3,\dots,2N_g$
Sum Entropy	Aggregates of neighborhood intensity values distinctions. $Sum\ Entropy = \sum_{i=1}^{N_g}\sum_{j=1}^{N_g} P_{x+y}(\kappa) log_2(P_{x+y}(\kappa)+\epsilon)$

The Gray Level Run Length Matrix (GLRLM) is is computed based on the number of connected voxels in the same intensity. GLRLM is characterized by an angle between pairs of voxels, θ. Elements (i,j) in the matrix represents the number of voxels with intensity i and run length j in a specified direction.

Mathematically, the GLRLM of an image with $Nx \times Ny$ dimensions and Ng different intensity levels is computed using the following equation

$$GLRM_\theta(i,j) = |\{(m,n): I(k,l) \epsilon Nb(m,n,j,\theta): I(k,l) = i\}| \forall\ i,j \in \{1,2,3,\dots,N_g\} \tag{2}$$

where $$Nb(m,n,j,\theta) = \begin{cases} \{(m+1,n),(m+2,n),\dots,(m+j,n)\} & if\ \theta = 0^\circ \\ \{(m+1,n+1),(m+2,n+2),\dots,(m+j,n+j)\} & if\ \theta = 45^\circ \\ \{(m,n+1),(m,n+2),\dots,(m,n+j))\} & if\ \theta = 90^\circ \\ \{(m-1,n-1),(m-2,n-2),\dots,(m-j,n-j)\} & if\ \theta = 135^\circ \end{cases}$$

Table S4: A summary of GLRM variable

Paramete	Description
GrayLevelNonuniformity	Measures the similarity of gray-level intensity values within an image. A lower GLN value indicates greater uniformity or similarity in intensity values across the image, implying less variation and a more consistent distribution of gray levels throughout the image. $GrayLevelNonuniformity = \frac{\sum_{i=1}^{N_g}\sum_{j=1}^{N_r} p(i,j\|\theta)^2}{N_r\theta}$
HighGrayLevelRunEmphasis	Quantifies the distribution of voxels with higher-intensity values. $HighGrayLevelRunEmphasis = \frac{\sum_{i=1}^{N_g}\sum_{j=1}^{N_r} p(i,j\|\theta) i^2}{N_r\theta}$ Where $N_r(\theta)$ is a number of runs in an image along angle θ and calculated as follows: $N_r(\theta) = \sum_{i=1}^{N_g}\sum_{j=1}^{N_r} p(i,j\|\theta), 1 \le N_r(\theta) \le N_p$
LongRunHighGrayLevelEmphasis	Quantifies joint distribution of voxels with higher intensity and long run length. $LongRunHighGrayLevelEmphasis = \frac{\sum_{i=1}^{N_g}\sum_{j=1}^{N_r} p(i,j\|\theta) i^2 j^2}{N_r\theta}$
LongRunLowGraylevelEmphasis	Measures sets of voxels with an identical intensity value.

	$LongRunLowGrayLevelEmphasis = \frac{\sum_{i=1}^{N_g}\sum_{j=1}^{N_r}\frac{p(i,j\|\theta)j^2}{i^2}}{N_r\theta}$
LowGrayLevelRunEmphasis	Estimate the distribution of images' lower intensity values $LowGrayLevelEmphasis = \frac{\sum_{i=1}^{N_g}\sum_{j=1}^{N_r}\frac{p(i,j\|\theta)}{i^2}}{N_r\theta}$
RunVariance	Measures variance of runs based on existing run length. $RunVariance = \sum_{i=1}^{N_g}\sum_{j=1}^{N_r}p(i,j\|\theta)(j-\mu)^2$ Where $\mu = \sum_{i=1}^{N_g}\sum_{j=1}^{N_r}p(i,j\|\theta)j$

The Gray Level Size Zone Matrix (GLSZM) quantifies intensity zones in an image. A zone is defined as some connected voxels with the same intensity level. The $(i,j)th$ element of GLSZM represents the number of zones with intensity i and size j seen in the image. Unlike the GLCM and GLRLM, there is no dependency on the θ to the generation of GLSZM, and thus, only a unique matrix will be calculated considering different directions.

Table S5: A summary of GLSZM variable

Paramete	Description
HighGraylevelzoneEmphasis	Measures the distribution of higher intensity size zones. $HighGraylevelzoneEmphasis = \frac{\sum_{i=1}^{N_g}\sum_{j=1}^{N_s}p(i,j)i^2}{N_z}$
GrayLevelVariance	Measures variance of intensity level based on existing zones. $GrayLevelVariance = \sum_{i=1}^{N_g}\sum_{j=1}^{N_s}p(i,j\|\theta)(j-\mu)^2$ Where $\mu = \sum_{i=1}^{N_g}\sum_{j=1}^{N_s}p(i,j\|\theta)j$
SizeZoneNonUniformity	Quantifies the variability of size zone volumes within an image. $SizeZoneNonUniformity = \frac{1}{N_z}\sum_{j=1}^{N_s}\sum_{i=1}^{N_g}p(i,j)^2$
SmallAreaEmphasis	Evaluates the distribution of small-size zones within a texture. Its higher value suggests a higher prevalence of smaller size zones and finer textures, highlighting the presence of intricate details and finer variations in the image texture. $SmallAreaEmphasis = \frac{1}{N_z}\sum_{i=1}^{N_g}\sum_{j=1}^{N_s}\frac{p(i,j)}{j^2}$
Zone Percentage	Quantifies the texture's coarseness based on the ratio between number of zones and number of voxels. $ZonePercentage = \frac{N_z}{N_p}$ Where N_z represents the number of zones in ROI and is calculated as $N_z = \sum_{i=1}^{N_g}\sum_{j=1}^{N_r}p(i,j)$
ZoneEntropy	Quantifies the uncertainty or randomness in the distribution of zone sizes and gray levels. $ZoneEntropy = \sum_{i=1}^{N_g}\sum_{j=1}^{N_s}p(i,j)log_2(p(i,j)+\varepsilon)$

The visualization of the Pearson Correlation Coefficient (PCC), Relative Percent Difference (RPD), and P-value for all 74 velocity informatics parameters between the two flow models is presented in Figure S6.

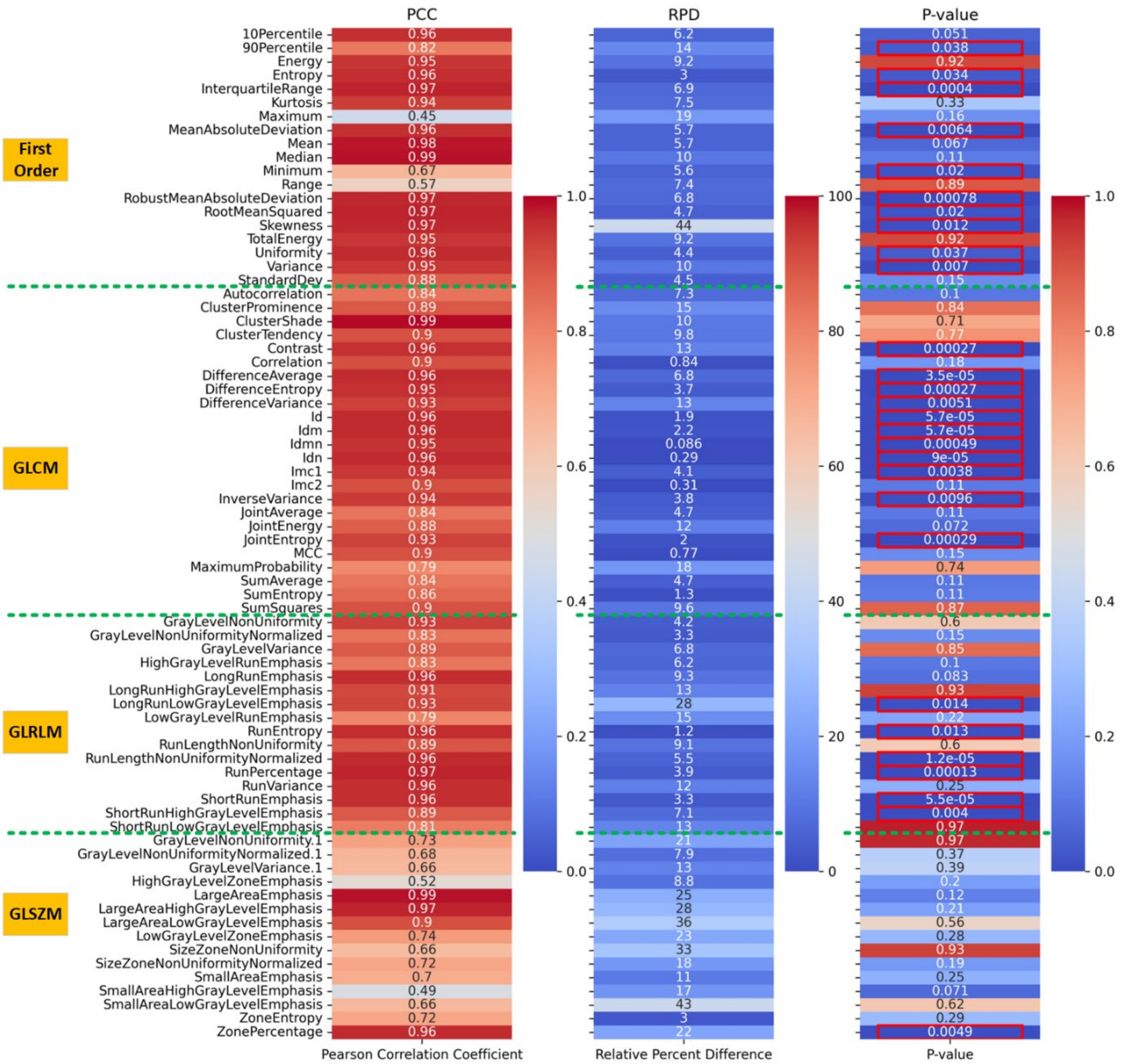

Figure S6: Heat maps depicting PCC, RPD, and P-values for all velocity informatics parameters, comparing Newtonian and non-Newtonian models. P-values, calculated using the paired T-test, highlight statistically significant differences (marked with red boxes) between the two models.

S4 - Linear regression and Bland-Altman of all hemodynamic parameters

Table 2 in the manuscript describes the linear regression (LR) slope and the essential parameters of the Bland-Altman (BA) analysis. Figures S6 and S7 illustrate the BA and LR plots of the seven parameters, respectively.

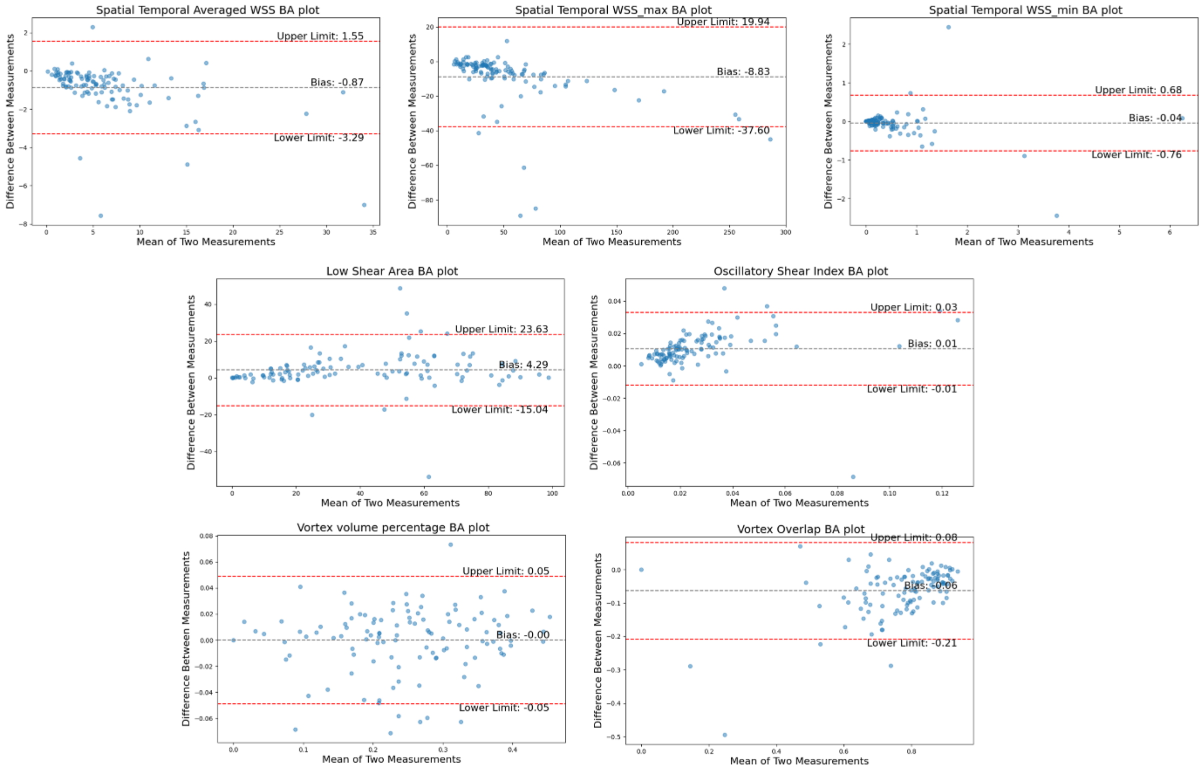

Figure S7: Demonstration of BA analysis for all seven hemodynamic parameters. The bias is calculated as non-Newtonian minus Newtonian.

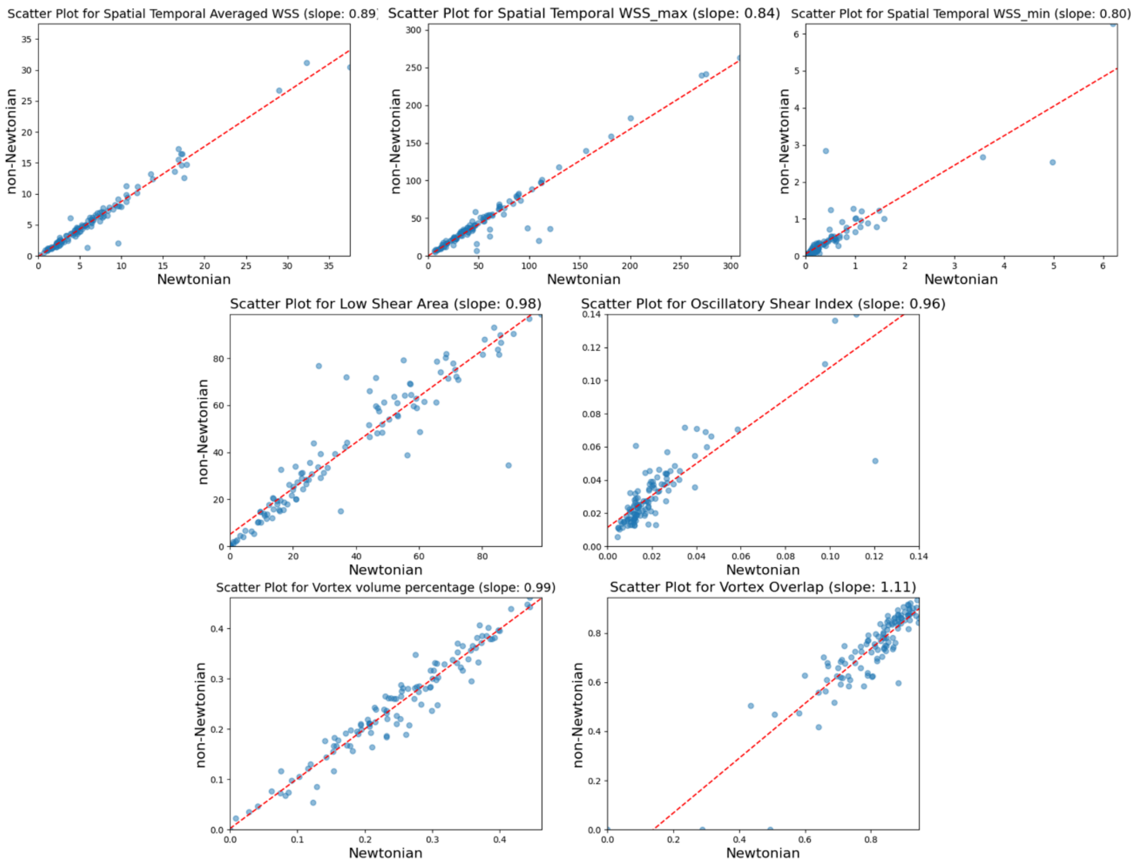

Figure S8: Demonstration of LR analysis for all seven hemodynamic parameters. The slope of each plot is included in the plot title.

S.5 Sensitivity of Outlet Boundary Conditions

Furthermore, as highlighted in other studies, different outlet boundary condition strategies can lead to significantly different results, with variations of up to 70% in the Newtonian model, as shown in Table S6. (Chnafa *et al.*, 2018a) Therefore, we also conducted a secondary study to investigate the potential effects of outlet boundary conditions with the Carreau-Yasuda model in 10 randomly selected cases. The strategy employed a local flow diversion method suggested by Chnafa et al. (Chnafa *et al.*, 2018b). In their method, the blood flow divides according to a power law equation at each bifurcation:

$$\frac{Q_1}{Q_2} = \left(\frac{D_1}{D_2}\right)^n \tag{3}$$

Upon finishing the simulation, the result was compared to the result of Carreau-Yasuda using the zero-pressure outlets. Table S6 summarizes the comparison of wall shear stress-derived parameters.

Table S6: A comparison of differences in hemodynamic parameters under two different outlet boundary conditions (zero-pressure outlet vs. a flow diversion scheme by Chnafa *et al.*). RPD is presented as (Mean ± SD). The slope represents the relationship between the Newtonian (x-axis) and non-Newtonian (y-axis) models.

Parameter	RPD (%)	Slope	PCC	Bias	Up-lim	Low-lim
STA-WSS (Pa)	37.26 ± 25.55	1.32	0.90	-6.59	5.11	-18.28
WSS-max (Pa)	70.45 ± 17.55	1.7	0.97	-86.52	41.53	-214.6
WSS-min (Pa)	69.11 ± 60.83	0.2	0.78	0.29	1.93	-1.36
LSA (%)	57.21 ± 53.41	1.09	0.94	-3.43	11.30	-18.16
OSI	61.28 ± 43.40	0.27	0.36	0.01	0.04	-0.02
RRT	72.21 ± 59.36	0.68	0.96	-0.63	1.70	-2.96
DVO	22.68 ± 20.31	0.79	0.25	0.15	0.46	-0.16
Vt/V	60.06 ± 38.47	0.72	0.68	0.14	0.26	0.02

S.6 Sensitivity of Velocity Informatics Metrics Using Two Additional Non-Newtonian Casson and HB Models

We also compared two additional non-Newtonian Casson and HB models with the Newtonian model. Figure S9 presents the PCC and RPD of velocity informatics parameters obtained from the Newtonian and Casson models, followed by a similar plot showing the PCC and RPD of velocity informatics parameters obtained from the Newtonian and HB model in Figure S10. Of note, the overall trend observed from Figures S9 and S10 is similar to Figure 2 in the manuscript. P-values were not calculated because this secondary study was only done for 10 randomly selected cases. In contrast, in Figure 2 in the manuscript, velocity informatics parameters were obtained from 112 cases, and thus, p-values were assessed and displayed as heat maps.

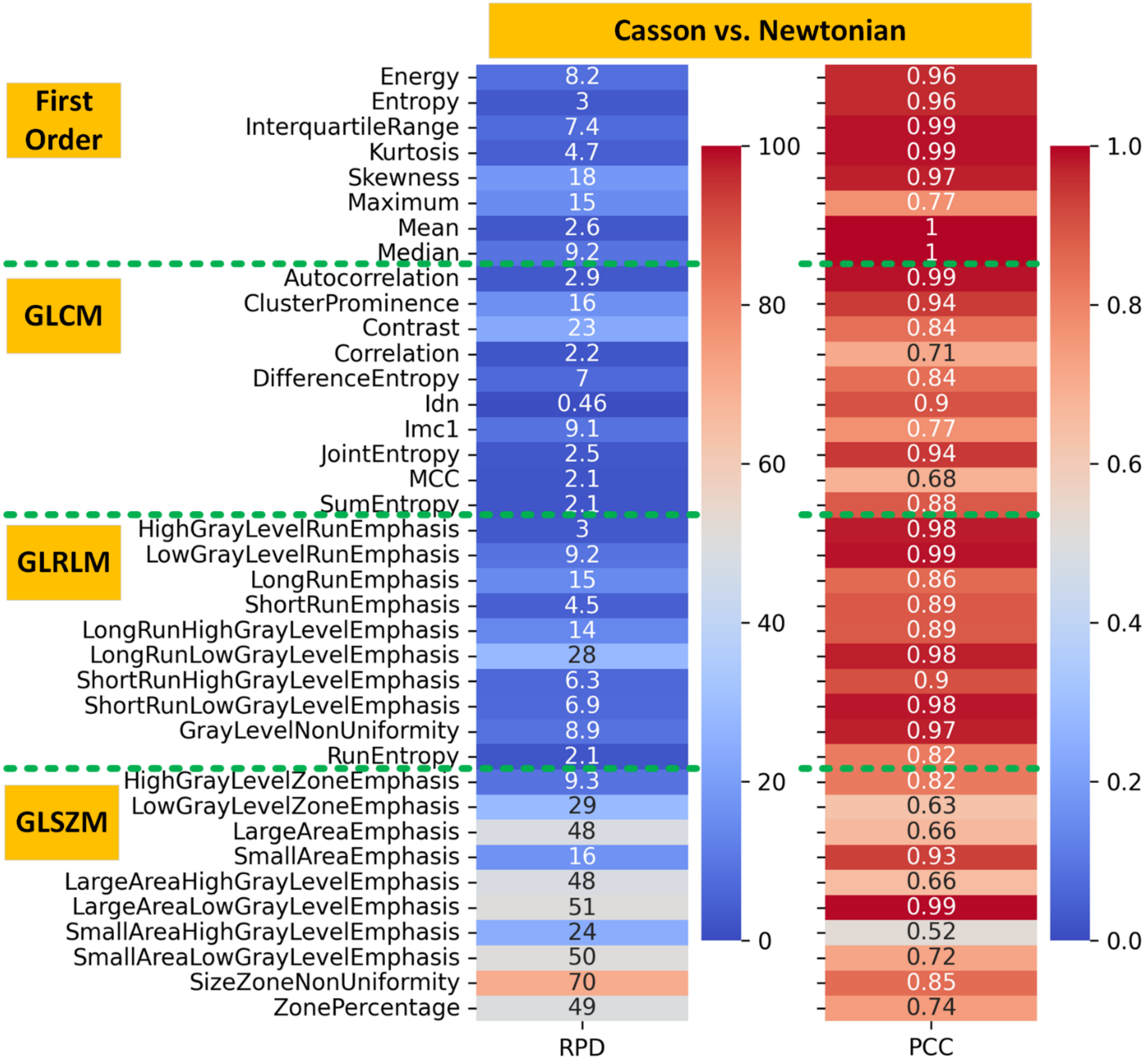

Figure S9: Heat maps depicting PCC and RPD for selected velocity informatics parameters, comparing Newtonian and non-Newtonian Casson models. Since the comparison was only conducted for 10 randomly selected cases, P-values were not calculated.

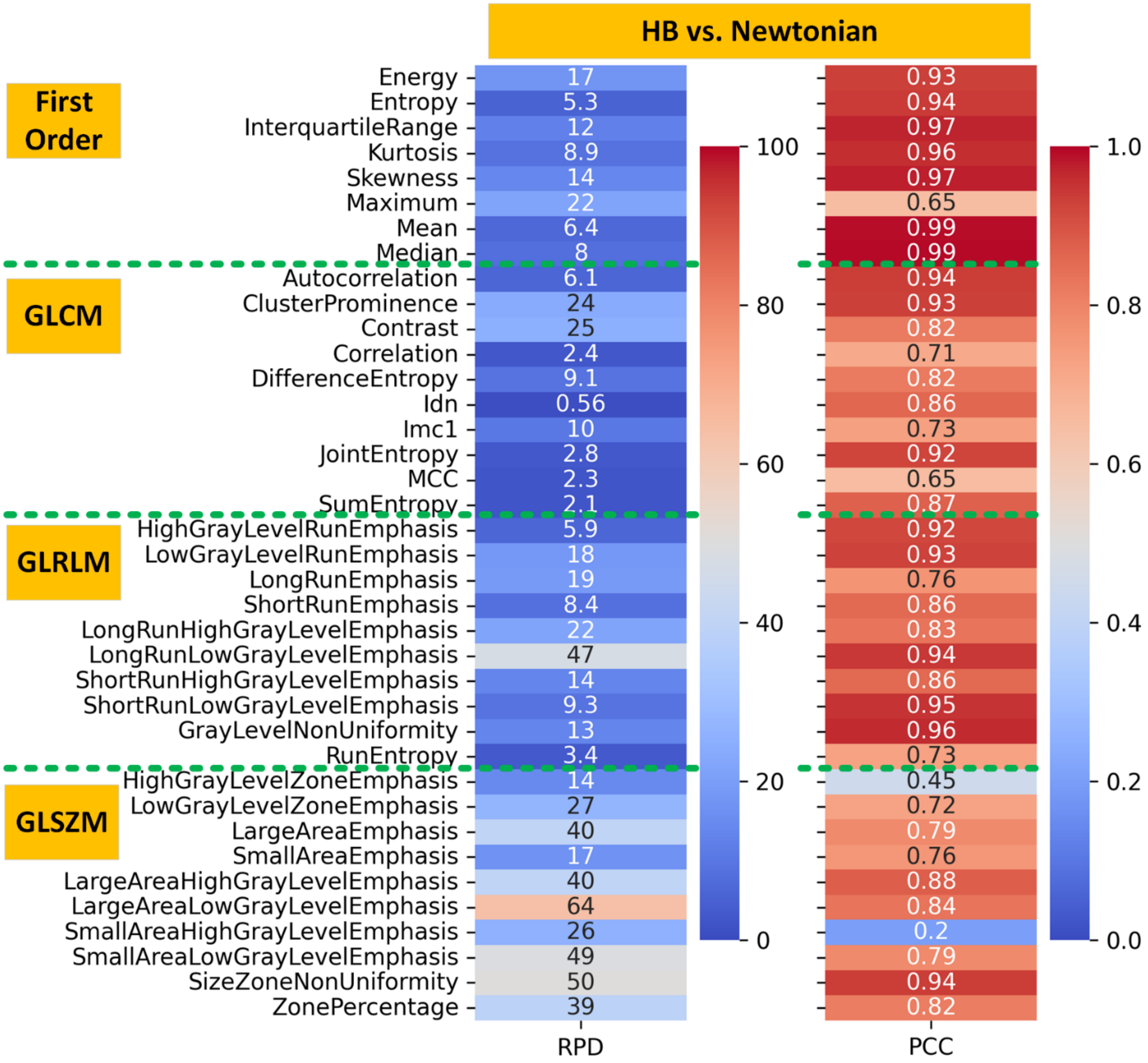

Figure S10: Heat maps depicting PCC and RPD for selected velocity informatics parameters, comparing Newtonian and non-Newtonian HB models. Since the comparison was only conducted for 10 randomly selected cases, P-values were not calculated.

Reference

Chnafa C, Brina O, Pereira V M and Steinman D A 2018a Better Than Nothing: A Rational Approach for Minimizing the Impact of Outflow Strategy on Cerebrovascular Simulations *American Journal of Neuroradiology* **39** 337

Chnafa C, Brina O, Pereira V M and Steinman D A 2018b Better Than Nothing: A Rational Approach for Minimizing the Impact of Outflow Strategy on Cerebrovascular Simulations *American Journal of Neuroradiology* **39** 337-43

Jiang J, Rezaeitaleshmahalleh M, Lyu Z, Mu N, Ahmed A, Md C S, Gemmete J and Pandey A J J o c t r 2023 Augmenting prediction of intracranial aneurysms' risk status using velocity-informatics: initial experience **16** 1153-65

Jiang J and Strother C M 2013 Interactive Decomposition and Mapping of Saccular Cerebral Aneurysms Using Harmonic Functions: Its First Application With "Patient-Specific" Computational Fluid Dynamics (CFD) Simulations *IEEE Transactions on Medical Imaging* **32** 153-64

Piccinelli M, Veneziani A, Steinman D A, Remuzzi A and Antiga L 2009 A Framework for Geometric Analysis of Vascular Structures: Application to Cerebral Aneurysms *IEEE Transactions on Medical Imaging* **28** 1141-55

van Griethuysen J J M, Fedorov A, Parmar C, Hosny A, Aucoin N, Narayan V, Beets-Tan R G H, Fillion-Robin J-C, Pieper S and Aerts H J W L 2017 Computational Radiomics System to Decode the Radiographic Phenotype *Cancer Research* **77** e104-e7
